# Ensemble-Based Descriptor Analysis of Flexible Odorants: Conformational Landscapes of Geraniol, Nerol, and Linalool

**DOI:** 10.1021/acs.jpca.6c04688

**Published:** 2026-10-14

**Authors:** Beatriz Cuadrado-Benavent, Juan J. Nogueira, David Ferro-Costas

**Affiliations:** † Department of Chemistry, 1190 Universidad Autónoma de Madrid, Calle Francisco Tomás y Valiente, 7, 28049 Madrid, Spain; ‡ Departamento de Química Física, 16722 Universidade de Santiago de Compostela, 15782 Santiago de Compostela, Spain; § Institute for Advanced Research in Chemical Sciences (IAdChem), 16780 Universidad Autónoma de Madrid, 28049 Madrid, Spain

## Abstract

Conformational flexibility remains a central challenge in the molecular modeling of odorants because thermally accessible structures may differ from the potential-energy minima identified in conformational searches. Here, we combine automated conformational exploration, multistructural thermodynamics, and descriptor-based statistical analysis to characterize the conformational ensembles of three flexible acyclic terpenic alcohols: geraniol, nerol, and linalool. The conformational searches identify large numbers of conformers (119, 88, and 83 for geraniol, nerol, and linalool, respectively), but only a subset of these structures contributes substantially to the finite-temperature equilibrium ensemble. Across the three systems, entropy-related contributions tend to shift the equilibrium toward more extended arrangements than those favored electronically, leading to Gibbs free-energy minima at 310 K that can differ from the corresponding potential-energy minima. Descriptor–energy correlation analyses further show that, among the descriptors considered, global shape exhibits the most consistent association with thermodynamic stabilization, rather than individual local hydroxyl-mediated contacts; decomposition of the Gibbs free energies indicates that this trend is predominantly vibrational in origin. Principal component and clustering analyses reveal that the ensembles are better described as continuous conformational landscapes than as sharply separated structural families. These results highlight the limitations of relying only on potential-energy minima and support the use of ensemble-based, descriptor-guided molecular modeling strategies for flexible odorants and related acyclic terpenoids.

## Graphical abstract

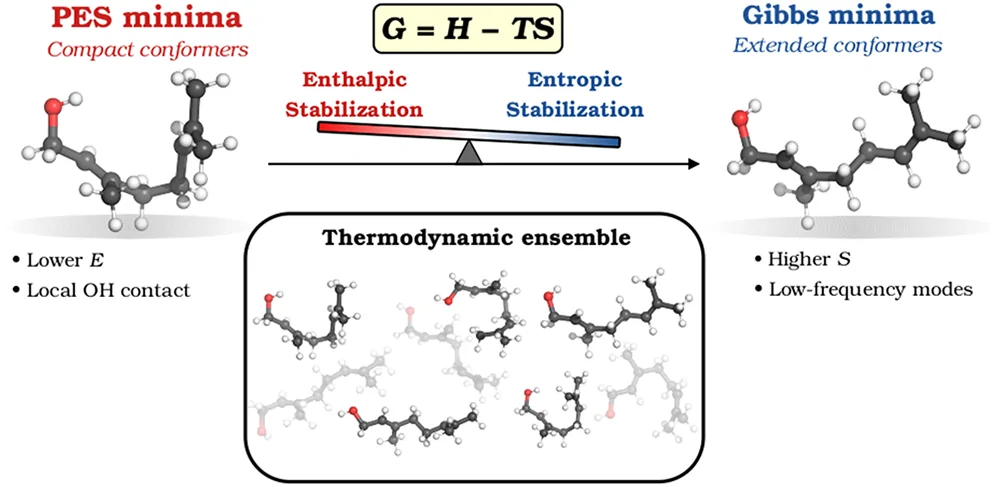

## 1 Introduction

Terpenoids constitute one of the largest and most structurally diverse families of natural products, encompassing thousands of compounds biosynthesized through the assembly and rearrangement of isoprene units.^1^ Their remarkable structural diversity underlies a broad range of biological and physicochemical functions and has motivated extensive applications in pharmaceuticals, cosmetics, food science, fragrances, and renewable-energy technologies.^2–5^ Monoterpenoids, containing two isoprene units and a C_10_ carbon framework, are among the most abundant members of this family and constitute major components of essential oils and odorant mixtures.^6^

Among monoterpenoids, terpenic alcohols such as geraniol, nerol, and linalool (C_10_H_18_O, Figure 1) are of particular interest because of their characteristic olfactory properties, diverse biological activities, and widespread use in fragrance, cosmetic, and flavor applications.^7–10^ For example, geraniol is a common fragrance terpene with a floral odor and has been reported in a range of consumer products, including deodorants, household products, and cosmetics.^11^

**Figure 1:**
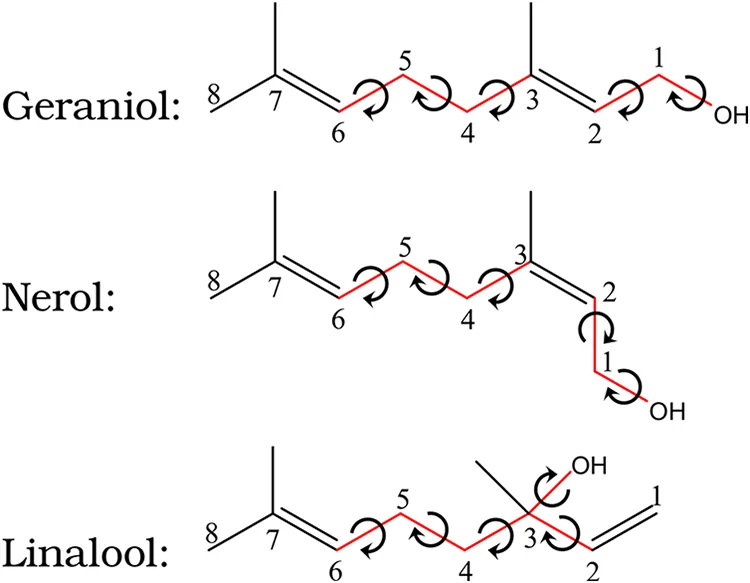
Molecular structures and atom numbering of the C_10_H_18_O terpenoids investigated in this work. Bonds highlighted in red correspond to the central bonds of the active torsional angles considered in the conformational exploration, and curved arrows indicate the associated internal rotations.

Geraniol, nerol, and linalool also represent prototypical examples of highly flexible acyclic monoterpenoids. Multiple internal degrees of freedom, together with low rotational barriers, generate highly multidimensional conformational landscapes containing numerous thermally accessible conformers with distinct structural, energetic, and thermodynamic characteristics. A similar conformational complexity has previously been observed in related terpene systems and flexible odorant molecules.^12^

Such conformational flexibility is not merely a structural feature but can have important functional consequences. Structural variations in flexible terpenoids may also influence chemical reactivity and broader biological behavior.^11^ Previous experimental and computational studies have shown that odorant recognition is sensitive not only to functional-group identity but also to the accessible conformational repertoire of the ligand.^13–15^ In particular, olfactory-receptor activation can depend on geometric constraints involving odorant extension and conformational accessibility.^12^ For example, conformationally restricted analogues of octanal revealed that semiextended conformations preferentially activate specific receptors, whereas more compact arrangements may behave as antagonists.^13,15^ These observations suggest that olfactory receptors may distinguish among different regions of an odorant conformational ensemble. Furthermore, increasing conformational flexibility has been proposed to broaden the accessible recognition space of odorants and potentially increase the number of receptors activated by a given molecule.^13^ Collectively, these findings highlight the importance of characterizing thermally accessible conformational ensembles when establishing realistic structure–function relationships in odorant systems.

If conformational ensembles play an important role in odorant recognition, then a key question is how these ensembles should be represented computationally. The conformers that minimize the potential-energy surface (PES) do not necessarily coincide with those that dominate the thermal equilibrium distribution at finite temperature. In flexible systems, equilibrium populations emerge from a subtle interplay among electronic stabilization, intramolecular interactions, and conformational entropy. Consequently, descriptions based exclusively on the PES global minimum may fail to capture thermodynamic behavior, and multistructural (MS) approaches provide a more realistic framework for flexible molecules. Despite the broad relevance of terpenic alcohols, the relationship between PES minima and thermodynamically dominant conformers remains insufficiently understood.^16,17^ In particular, the role of conformational entropy in reshaping equilibrium populations and the structural factors governing conformational organization remain largely unexplored.

In the present work, we perform a comprehensive conformational and thermodynamic study of the C_10_H_18_O terpenoids geraniol, nerol, and linalool. For the latter, only (*R*)-linalool (licareol) was considered since the two enantiomers are related by reflection and therefore share identical electronic energies, vibrational frequencies, and partition functions in an achiral environment. Their conformational landscapes are explored using the TorsiFlex program,^18,19^ while MS thermodynamic treatments are used to evaluate conformer populations and temperature effects.^20–22^ To characterize the structural organization of the resulting ensembles, geometric descriptors are combined with descriptor–energy correlation analyses, principal component analysis (PCA),^23^ and clustering techniques commonly employed in unsupervised molecular data analysis.^24–26^ Particular attention is devoted to understanding how global molecular shape, molecular compactness, local hydroxyl-mediated interactions, and entropy-related contributions collectively shape thermal equilibrium conformational ensembles. The present study therefore aims to provide a physically motivated description of the conformational behavior of representative terpenic alcohols and establishes a framework for future studies of odorant recognition and ensemble-based molecular modeling.

## 2 Computational Methods

The computational workflow combined automated conformational exploration, MS thermodynamics, and descriptor-based statistical analysis. Conformational landscapes were explored with TorsiFlex, and the resulting minima were used to compute MS partition functions and equilibrium populations. The structural organization of the ensembles was characterized through geometric descriptors describing molecular extension, global shape, and local intramolecular contacts, followed by descriptor–energy correlation analyses, PCA, and clustering analyses.

Unless otherwise stated, relative energies refer to the lowest-energy conformer of the corresponding quantity. In comparisons between different levels of theory or thermodynamic approximations, all quantities are instead referred to a common reference, namely, the conformer minimizing the electronic energy at the MPWB1K/6-31+G­(d,p) level, so that the comparison is not affected by changes in the identity of the minimum.

### 2.1 Conformational Search with TorsiFlex

The conformational landscapes of geraniol, nerol, and linalool were explored using the TorsiFlex program,^18,19^ which combines systematic and stochastic exploration of the multidimensional torsional space associated with the active internal rotations. For each terpenoid, five conformationally active torsional coordinates were considered; the corresponding central bonds are highlighted in Figure 1. Dihedral angles involving unsaturated central bonds were excluded because rotation around double bonds does not constitute an accessible conformational degree of freedom. Torsions associated with terminal methyl groups were also omitted since their rotation does not lead to distinct conformers. As discussed in Section 1, only (*R*)-linalool (licareol) was explicitly explored since the results are equally valid for the (*S*) enantiomer.

TorsiFlex explores conformational space through a structure-generation strategy rather than through a conventional relaxed torsional scan with a fixed angular step size. Starting from a trial geometry provided in Z-matrix format, new trial structures were generated by replacing the dihedral angles associated with the selected torsions, while the remaining internal coordinates were taken from the reference or previously optimized structures. The initial exploration combined two complementary stages. First, a preconditioned systematic search was performed by assigning chemically intuitive *gauche* ^+^, *anti*, and *gauche* ^–^ arrangements to the selected torsional coordinates. Second, stochastic torsional sampling was used to generate additional trial structures and to explore regions of the multidimensional conformational space that were not necessarily covered by the preconditioned search.

The resulting trial structures were optimized at the HF/3-21G level, and redundant conformers were discarded using the torsional similarity and redundancy tests implemented in TorsiFlex. The corresponding tolerances, together with the parameters governing the stochastic stage and its convergence, are collected in the Supporting Information (SI). Structures with imaginary frequencies were also excluded. For geraniol and nerol, torsional symmetry was exploited during the search: only one member of each mirror-related torsional pair was explicitly explored, and the corresponding symmetry-related conformer was generated automatically after optimization.

After completion of the low-level exploration, all distinct conformers were reoptimized at the MPWB1K/6-31+G­(d,p) level. MPWB1K was selected because it was parametrized for thermochemistry and noncovalent interactions^27,28^ and has been successfully employed with the 6-31+G­(d,p) basis set in previous conformational studies of flexible oxygenated molecules.^29,30^ Harmonic frequency calculations were then carried out at the same level of theory to confirm the nature of high-level stationary points and to compute the partition functions required for the MS thermodynamic treatment described in the next subsection.

It is worth emphasizing that HF/3-21G is used exclusively to generate candidate structures: no information obtained at this level is retained, and every stored structure is reoptimized at a higher level. This two-level strategy is the one for which TorsiFlex was designed and validated,^18,19^ and it has also been employed by some of us for flexible acyclic alcohols.^29,30^ Although HF lacks electron correlation, the 3-21G basis set partly compensates this deficiency through error cancellation, and this combination describes intramolecular hydrogen bonding better than traditional semiempirical methods,^31^ which is relevant here because the compact conformers of these terpenic alcohols are stabilized by local hydroxyl-mediated contacts. Although a two-level protocol cannot formally guarantee that every high-level minimum has a counterpart on the low-level surface, the bias of this particular choice operates in the opposite direction: HF tends to generate more minima than correlated methods, with the spurious ones disappearing upon high-level reoptimization.

The generation of trial structures, preparation and management of low- and high-level electronic-structure calculations, duplicate detection, frequency-based filtering, and the application of symmetry operations were automatically handled within the TorsiFlex workflow. The electronic-structure calculations managed by TorsiFlex were performed with the Gaussian 16 program.^32^ All electronic-structure calculations were performed in the gas phase (in vacuo) without any implicit or explicit treatment of a surrounding medium.

Since MPWB1K does not include an explicit dispersion correction, the relative energies of all conformers were also evaluated through single-point calculations on the MPWB1K geometries at the ωB97X-D/def2-TZVP and DLPNO-CCSD­(T)/def2-TZVP levels. These results are reported in the SI; unless otherwise stated, all results discussed below correspond to the MPWB1K/6-31+G­(d,p) level.

### 2.2 Multistructural Thermodynamics

#### 2.2.1 Multistructural Harmonic-Oscillator (MS-HO) Approximation

In the conventional one-well (1W) approximation, only the conformer associated with the global minimum of the PES is considered in the calculation of the partition function:

1 $$Q^{1W}\left(T\right)=Q_{0}^{\mathrm{RRHO}}\left(T\right)\times \exp\left[-\frac{E_{0}^{\mathrm{el}}+E_{0}^{\mathrm{ZPE}}}{k_{B}T}\right]$$

where *k*_B_ is the Boltzmann constant, *Q*_0_^RRHO^(*T*) is the rigid-rotor harmonic-oscillator (RRHO) partition function of the lowest-energy conformer, and *E*_0_^el^ and *E*_0_^ZPE^ are its electronic and zero-point energies, respectively. Within this approximation, the corresponding Gibbs free energy is given by

2 $$G\left(T\right)=-k_{B}T\;\ln Q^{1W}\left(T\right)=\left(E_{0}^{\mathrm{el}}+E_{0}^{\mathrm{ZPE}}\right)-k_{B}T\;\ln\left[Q_{0}^{\mathrm{RRHO}}\left(T\right)\right]$$

The translational and the electronic partition-function contributions are omitted from eqs 1 and 2 since only relative Gibbs free energies between conformers of the same molecule are considered. These terms are identical for all conformers and cancel upon taking free-energy differences. Thus, the relative Gibbs free energies are determined by the zero-point-inclusive electronic energies and the rotational and vibrational contributions.

For flexible molecules containing multiple thermally accessible conformers (*J*), a multistructural treatment becomes necessary. Within the multistructural harmonic-oscillator (MS-HO) approximation, the molecular partition function is written as a sum over all accessible conformers:^20–22^

3 $$Q^{\mathrm{MS}}\left(T\right)=\sum_{j=0}^{J-1}Q_{j}^{1W}\left(T\right)$$

where *Q*_*j*_^1W^(*T*) is the one-well partition function associated with conformer *j*, obtained analogously to eq 1. Within this approximation, the relative population of a given conformer *j*, χ_*j*_(*T*), is then given by

4 $$\chi_{j}\left(T\right)=\frac{Q_{j}^{1W}\left(T\right)}{Q^{\mathrm{MS}}\left(T\right)}$$

The sum in eq 3 runs over the complete set of conformers located for each system, so that the two members of a mirror-related pair enter as separate terms; this is equivalent to assigning a multiplicity of two to one representative of each pair, which is how the degeneracy is handled in practice. No further symmetry factor is required: all of the conformers of the three terpenoids belong to the *C*_1_ point group, with the single exception of one *C*_*s*_ structure of geraniol, and the rotational symmetry number is therefore unity in every case.^33^

In practical applications, empirical scaling factors are frequently applied to harmonic vibrational frequencies in order to partially compensate for anharmonic effects.^34,35^ In the present work, all harmonic frequencies entering the RRHO partition functions were scaled by λ^ZPE^ = 0.951, the factor recommended by Alecu et al.^36^ for thermochemical applications at the MPWB1K/6-31+G­(d,p) level.

#### 2.2.2 MS Torsional Approximation with Coupled Potential

The MS-HO treatment described above relies on a harmonic description of all vibrational modes, including the softest torsions, and a uniform frequency scale factor may not account for hindered internal rotation. To assess the impact of this approximation, the partition functions were additionally evaluated within the multistructural torsional approximation with coupled potential [MS-T­(C)],^20,37^ which replaces the harmonic treatment of the torsional modes by a hindered rotor description including couplings among the torsions and between the torsions and the remaining degrees of freedom. These calculations were performed using the MSTor program^38^ with input files generated by TorsiFlex. Unless otherwise stated, all results reported here correspond to the MS-HO treatment; the MS-T­(C) calculations are used to verify their robustness.

It should be noted that the MS-T­(C) treatment involves eight torsional coordinates rather than the five active ones used in the conformational search. The three methyl rotations, which were excluded from the exploration because they do not generate distinct conformers, do contribute to the torsional partition function and are therefore included here, each with a local periodicity of three. The remaining parameters were those generated by TorsiFlex from the final conformer sets, and the same frequency scale factor as that in the MS-HO calculations was applied.

### 2.3 Geometric Descriptors of the Conformational Ensemble

To obtain a physically interpretable representation of the conformational ensembles, a set of geometric variables was introduced (Table 1). These quantities were grouped into two categories according to the information that they provide: (i) descriptors associated with global molecular extension and molecular shape and (ii) descriptors characterizing local hydroxyl-mediated interactions and intramolecular contacts. All of these descriptors are invariant under reflection. Consequently, they take identical values for each mirror-related pair of torsional conformers in geraniol and nerol. Likewise, the descriptor-based analyses obtained for (*R*)-linalool are identical with those that would be obtained for (*S*)-linalool.

**Table 1:** Descriptors Employed for the Characterization of the Conformational Ensembles

descriptor		definition	interpretation
(D_1_)	*d*_O–C_far__	O-farthest terminal C distance	O-to-periphery extension
(D_2_)	*R*_g_	heavy-atom radius of gyration	global spatial extent
(D_3_)	κ^2^	relative shape anisotropy	shape anisotropy
(D_4_)–(D_5_)	*d*_O···π,*i*_	O···C=C centroid distance	OH proximity to unsaturated region
(D_6_)–(D_7_)	*d*_H_O_···π,*i*_	H_O_···C=C centroid distance	potential OH···π interaction
(D_8_)	*d*_O···H_C_,min_	shortest O···H–C distance	local intramolecular contacts
(D_9_)	*c*_O_	oxygen coordination descriptor	local molecular packing
(D_10_)	*c*_H_O__	H_O_ coordination descriptor	directional local interactions
(D_11_)	θ_O–C_α_–C_far__	O–C_α_–C_far_ angle	OH orientation

#### 2.3.1 Global-Shape Descriptors

The extension of each conformer from the oxygen atom toward the molecular periphery was characterized by the distance to the farthest terminal carbon (C_far_), *d*_O–C_far__, with C_far_ selected independently for each conformer. In most conformers, C_far_ corresponds to one of the two methyl carbons of the isopropylidene moiety, −CH=C­(CH_3_)_2_. In some nerol conformers, however, the largest O···C separation instead involves the methyl substituent attached to the C=C bond proximal to the hydroxyl group.

The global size of the conformers was also characterized by the heavy-atom radius of gyration,^39^

5 $$R_{g}={\left[\frac{1}{N_{h}}\sum_{a=1}^{N_{h}}{\left|r_{a}-r_{c}\right|}^{2}\right]}^{1/2}$$

where *N*_h_ is the number of heavy atoms, **r**_*a*_ denotes the position vector of atom *a*, and **r**_c_ is the geometric center of the heavy-atom framework. No mass weighting was applied.

Whereas *d*_O–C_far__ is a single pairwise distance between the oxygen atom and the farthest terminal carbon in each conformer, *R*_g_ accounts for the spatial distribution of the entire heavy-atom skeleton. Larger *R*_g_ values correspond to a more spatially extended heavy-atom framework, whereas smaller values correspond to a more compact one. This global measure is useful when a single O···C distance does not fully capture the overall molecular size.

The corresponding directional information is contained in the gyration tensor,^39^

6 $$S=\frac{1}{N_{h}}\sum_{a=1}^{N_{h}}\left(r_{a}-r_{c}\right){\left(r_{a}-r_{c}\right)}^{T}$$

whose trace is related to the radius of gyration through *R*_g_^2^ = Tr­(**S**) = λ_1_ + λ_2_ + λ_3_, where λ_1_–λ_3_ are the eigenvalues of **S**. These eigenvalues can be used to quantify the anisotropy of the molecular shape through the relative shape anisotropy parameter κ^2^:

7 $$\kappa^{2}=\frac{3}{2}\frac{\lambda_{1}^{2}+\lambda_{2}^{2}+\lambda_{3}^{2}}{{\left(\lambda_{1}+\lambda_{2}+\lambda_{3}\right)}^{2}}-\frac{1}{2}$$

Small values of κ^2^ indicate a more isotropic distribution of the heavy-atom skeleton, whereas larger values indicate increasingly anisotropic or elongated shapes.^39^

Thus, *d*_O–C_far__, *R*_g_, and κ^2^ describe complementary aspects of molecular geometry.

#### 2.3.2 Local Hydroxyl-Interaction Descriptors

Several descriptors associated with local hydroxyl-mediated interactions and intramolecular contacts were introduced in order to characterize folded conformations and local packing effects. The distances between the oxygen atom and the centroids of the two C=C double bonds, *d*_O···π_1__ and *d*_O···π_2__, together with the corresponding distances from the hydroxyl hydrogen atom (hereinafter, H_O_), *d*_H_O_···π_1__ and *d*_H_O_···π_2__, were computed to characterize the proximity and orientation of the hydroxyl group with respect to the unsaturated regions of the molecule. Including both O···π and H_O_···π distances allows distinguishing conformers in which the hydroxyl group is spatially close to a C=C unit from those in which the O–H bond is also oriented toward that region. These descriptors provide geometric information regarding possible local OH···π contacts and folded molecular arrangements.

The minimum distance between the oxygen atom and hydrogen atoms bonded to carbon atoms, *d*_O···H_C_,min_, was also evaluated as a descriptor of short intramolecular contacts characteristic of compact molecular arrangements. To avoid trivial local interactions, hydrogen atoms attached to the carbon atom directly bonded to the hydroxyl group (hereinafter, C_α_) were excluded from the calculation.

To characterize the local environment around the hydroxyl group, oxygen- and hydrogen-centered continuous descriptors (*c*_O_ and *c*_H_O__) inspired by the coordination-number formalism implemented in PLUMED 2 were introduced:^40^

8 $$c_{X}=\sum_{i\neq X}\frac{1-{\left(r_{X,i}/r_{0}\right)}^{8}}{1-{\left(r_{X,i}/r_{0}\right)}^{16}}$$

where *r*_X,*i*_ denotes the distance between atom X and atom *i*, and *r*_0_ = 3.0 Å defines the characteristic distance over which neighboring atoms contribute appreciably to the descriptor. The switching function approaches 1 for atoms located close to X and smoothly decays toward 0 as the distance increases. Consequently, larger values of *c*_X_ indicate a more crowded local environment around atom X, whereas smaller values correspond to a more exposed or less locally packed environment. The value of *r*_0_ = 3.0 Å was chosen to describe short-range intramolecular contacts around the hydroxyl group, including contacts within the typical distance range relevant for local packing and weak noncovalent interactions in folded conformers.

The sum in eq 8 runs over all remaining atoms, without excluding those covalently bonded or topologically close to X. The contributions of these neighbors are essentially independent of the conformation, being fixed by the local covalent connectivity, and therefore, they enter the descriptor as an approximately constant offset that does not affect its conformational variability within a given molecule. They do, however, shift the absolute values of *c*_O_ and *c*_H_O__ differently in each system since the carbinol carbon is tertiary in linalool and primary in geraniol and nerol. These two descriptors are therefore meaningful for comparing conformers within a given system, rather than across systems.

Finally, the orientation of the hydroxyl group relative to the molecular framework was characterized by the angle θ_O–C_α_–C_far__, defined by the oxygen atom, C_α_, and C_far_.

### 2.4 Statistical Analysis of Conformational Descriptors

The geometric descriptors defined above were used to examine both their individual relationships with thermodynamic stability and the multidimensional organization of the conformational ensembles. Because reflection leaves all of the descriptors in Table 1 unchanged, mirror-related conformers require particular consideration in these analyses. For geraniol and nerol, mirror-related conformers are retained as separate contributions to the partition function, whereas only one representative of each mirror-related pair was retained for the descriptor-based statistical analyses since the two members yield identical descriptor vectors and therefore constitute duplicate observations.

#### 2.4.1 Descriptor–Energy Correlation Analysis

To evaluate the relationship between conformer geometry and thermodynamic stability, the relative Gibbs free energies at 310 K, *G*_rel_(310), were analyzed as a function of each geometric descriptor defined in Table 1.

Least-squares linear regressions were used to quantify approximate linear trends using the coefficient of determination, *R* ^2^, and the *p*-value for testing the null hypothesis of a zero slope.^41^ In addition, Spearman rank correlation coefficients, ρ_s_, were computed to assess monotonic relationships without assuming linearity, along with the corresponding *p*-values for testing the null hypothesis of no monotonic association.^42^

These analyses were used to identify the descriptors showing the most consistent one-dimensional relationships with thermodynamic stabilization across the three terpenoids. Descriptor relevance was assessed from both the magnitude of the correlation metrics and the corresponding *p*-values.

#### 2.4.2 Principal Component Analysis

PCA^23^ was employed to identify dominant collective variables within the conformational ensembles and reduce the dimensionality of the descriptor space (Table 1). Prior to PCA, all descriptors were standardized to a zero mean and unit variance. Principal components (PCs) were obtained via singular value decomposition^43^ of the standardized descriptor matrix. The number of PCs retained for subsequent analyses was selected individually for each system to account for at least 80% of the total variance, a criterion that retained three PCs in the three systems.

#### 2.4.3 Clustering Analysis

Clustering analyses were subsequently performed in the reduced PCA space. *K*-means clustering^24–26^ was explored using the silhouette^44^ and Davies–Bouldin^45^ indices, whereas Gaussian mixture models (GMMs)^24^ were evaluated using the Bayesian information criterion (BIC).^46^ Solutions containing 2–20 groups were examined for each system. The GMM analysis employed a shared covariance matrix (covariance_type = 'tied'), so that the components represent comparable regions of the continuous descriptor space rather than intrinsically distinct structural families. All PCA and clustering analyses were performed with scikit-learn.^47^ Details of the model comparison, initialization tests, and clustering diagnostics are provided in the SI.

## 3 Results and Discussion

This section discusses the conformational, thermodynamic, and statistical analyses performed for geraniol, nerol, and linalool.

### 3.1 Conformational Exploration

The conformational spaces of geraniol, nerol, and linalool were explored through the combined systematic–stochastic strategy implemented in TorsiFlex. Owing to the presence of multiple rotatable bonds and relatively shallow torsional potentials, all three systems exhibit highly multidimensional PESs characterized by numerous thermally accessible local minima.

If three preferred orientations (*gauche* ^+^, *anti*, and *gauche* ^–^) are considered for each of the five active torsions, a simple combinatorial count gives up to 3^5^ = 243 possible torsional arrangements. In practice, steric repulsions make some combinations unfavorable or inaccessible, and rotations involving bonds adjacent to unsaturated fragments are partially restricted.

At the HF/3-21G level, the conformational exploration yielded full ensembles of 159, 126, and 173 conformers for geraniol, nerol, and linalool, respectively, after accounting for the mirror-related counterparts generated through torsional enantiomerism, where applicable. Following MPWB1K/6-31+G­(d,p) reoptimization and validation, these ensembles were reduced to 119, 88, and 83 conformers, respectively. This reduction reflects the tendency of HF to generate additional spurious minima that disappear at the higher level and indicates that the torsional space was oversampled rather than underexplored during the prescreening stage.

Because torsional enantiomerism is present in geraniol and nerol, the final high-level ensembles correspond to 60, 44, and 83 symmetry-unique conformers for geraniol, nerol, and linalool, respectively (Table 2). For geraniol, 59 of these conformers have a distinct mirror-related counterpart, whereas one conformer has *C*_*s*_ symmetry and is its own mirror image, giving a full ensemble of 119 conformers (59 × 2 + 1). The 44 symmetry-unique nerol conformers each have a mirror-related counterpart, giving a full ensemble of 88 conformers. For (*R*)-linalool, reflection generates the opposite enantiomer rather than another conformer of the same stereoisomer, and no symmetry expansion was therefore applied. Accordingly, the thermodynamic calculations use the full ensembles of 119, 88, and 83 conformers (Sections 3.2 and 3.3), whereas the descriptor-based statistical analyses (Sections 3.4 and 3.5) use the corresponding symmetry-unique sets of 60, 44, and 83 conformers for geraniol, nerol, and linalool, respectively, thereby avoiding the inclusion of mirror-related structures as duplicate statistical observations.

**Table 2:** Number of Symmetry-Unique Conformers Identified in the Conformational Search (*N*_unique_) and the Total Number of Conformers after Application of Torsional Enantiomerism (*N*_conf_)

	geraniol	nerol	linalool
*N*_unique_	60	44	83
*N*_conf_	119	88	83

Previous conformational information for these acyclic terpenic alcohols is scarce and unevenly distributed among the three systems. For linalool, Nguyen et al. performed a quantum-chemical conformational search to support microwave spectroscopic measurements, starting from 167 force-field-preselected geometries and identifying 54 energy minima at the MP2/6-311++G­(d,p) level; under molecular-beam conditions, only the lowest-energy conformer was experimentally assigned.^48^ An earlier conformational analysis of linalool, carried out in a different context using a Monte Carlo/MM3 protocol, reported 88 low-energy conformers.^49^ In contrast, previous studies involving geraniol and nerol have mainly addressed specific conformational preferences, spectroscopic observables, or ion–molecule reaction properties rather than exhaustive conformational enumeration. To the best of our knowledge, no comparable conformational study reporting explicit numbers of conformers for geraniol or nerol has been published. The conformer sets obtained here therefore provide a consistent basis for comparing the conformational complexity of geraniol, nerol, and linalool within a common computational protocol.

### 3.2 Effective Size of the Thermodynamic Ensemble

Despite the large number of conformers identified, not all structures contribute equally to the thermodynamic behavior of the system. In order to quantify the effective size of the thermal equilibrium ensemble, the minimum number of conformers required to recover 90% of the total partition function (*N*_90_) was evaluated as a function of the temperature. For each temperature, conformers were ranked according to their equilibrium populations, eq 4, and progressively accumulated until reaching 90% of the MS partition function. Figure 2 summarizes the percentage of conformers required for this purpose.

**Figure 2:**
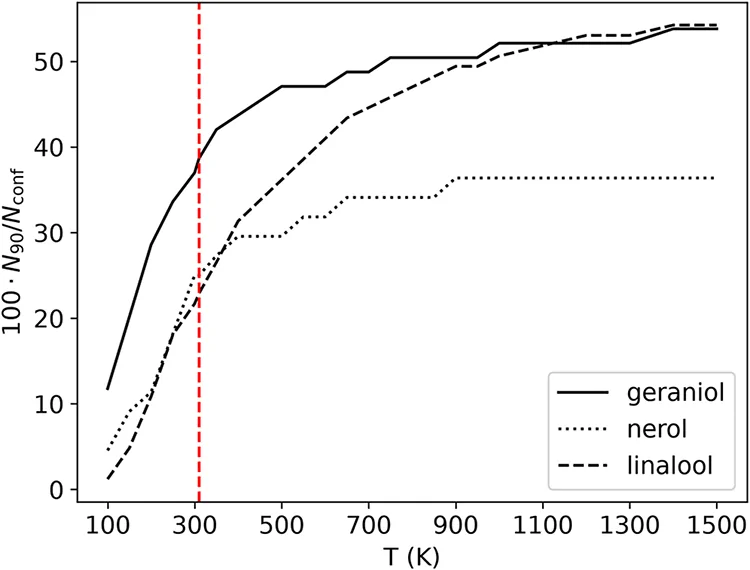
Minimum percentage of conformers required to account for 90% of the MS partition function, *Q* ^MS^, for the investigated terpenic alcohols. *N*_conf_ is the total number of conformers identified. *N*_90_ is the minimum number of conformers that, when ranked by decreasing equilibrium population, accumulate 90% of *Q* ^MS^ at a given temperature. The vertical dashed line marks 310 K.

At low temperatures, only a limited fraction of the conformational space contributes appreciably to the equilibrium ensemble. Under these conditions, the population is concentrated in the most favorable low-energy regions of the conformational landscape, and relatively few conformers are sufficient to recover most of the partition function. As temperature increases, additional conformers become thermally populated, and the effective ensemble size grows, although the extent of this thermal broadening differs markedly among the three terpenic alcohols.

Geraniol displays the broadest equilibrium ensemble over most of the temperature range. At human body temperature (310 K), it requires approximately 39% (*N*_90_ = 46) of the identified conformers to account for 90% of the MS partition function, indicating that its thermodynamic population is distributed over a relatively large number of structures. Nerol and linalool require smaller fractions at the same temperature, about 25% (*N*_90_ = 22) and 23% (*N*_90_ = 19), respectively, showing that their equilibrium populations are more concentrated within a reduced subset of conformers under near-physiological conditions.

The temperature dependence also reveals different ensemble-broadening patterns. Nerol reaches a comparatively low plateau, requiring only about one-third of its conformers even at high temperature. In contrast, linalool shows a more gradual increase with the temperature and eventually approaches the high-temperature fraction observed for geraniol. Thus, the effective thermal ensemble is not determined simply by the total number of PES minima but by how the MS partition function is distributed among them as temperature changes.

It should be noted that the temperature range in Figure 2 extends well beyond the conditions under which these compounds are chemically stable and is included only for comparative purposes. All subsequent results and discussions refer exclusively to 310 K.

### 3.3 From Electronic-Energy Minima to Thermodynamic Stabilization

#### 3.3.1 Structural and Thermodynamic Comparison of the Minima

Inspection of the conformers with the lowest electronic energies, *E* ^el^, and lowest Gibbs free energies at 310 K, *G*(310), reveals a common trend across the three investigated terpenoids. In all cases, the PES minima adopt compact or semifolded arrangements, whereas the Gibbs free-energy minima correspond to more extended molecular structures (Figure 3). As quantified in Table 3, the *G*(310) minima exhibit larger *d*_O–C_far__ and *R*_g_ values than the corresponding PES minima, pointing to an association between molecular extension and thermodynamic stabilization at 310 K.

**Figure 3:**
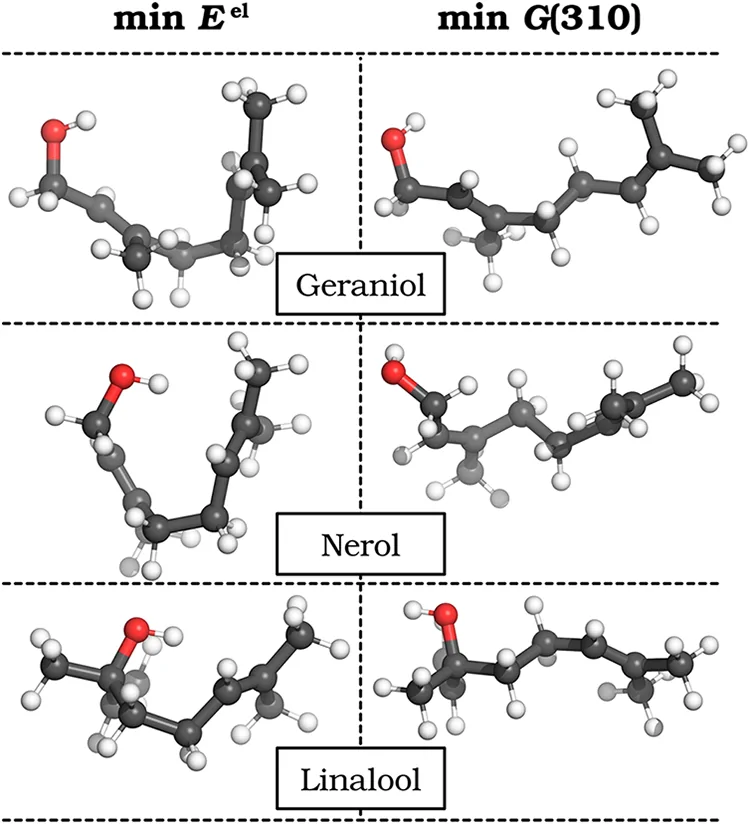
Conformer structures corresponding to the electronic-energy minima (left) and Gibbs free-energy minima at 310 K (right) for the investigated terpenoids.

**Table 3:** Relative Electronic Energies, Gibbs Free Energies at 310 K, Equilibrium Populations at 310 K, *R*_g_, and *d*_O–C_far__ Values for the Conformers Corresponding to the Minima in *E* ^el^ and *G*(310)^a^

molecule	minimum	type	*R*_g_	*d*_O–C_far__	*E*_rel_^el^	*G*_rel_(310)	population (%)
geraniol	*E*	semifolded	2.56	6.23	0.00	1.21	1.9
	*G*(310)	extended	3.05	8.78	0.51	0.00	13.8
nerol	*E*	semifolded	2.28	4.69	0.00	0.99	5.9
	*G*(310)	extended	2.86	7.87	1.80	0.00	29.1
linalool	*E*	semifolded	2.34	4.24	0.00	0.26	10.3
	*G*(310)	extended	2.73	6.64	1.53	0.00	15.8

a Energies are given in kcal mol^–1^, and distances are in Å.

This stabilization can be rationalized from the balance between electronic energy and vibrational entropy. Compact and semifolded conformers are electronically favored because they allow shorter local intramolecular contacts, including possible hydroxyl-mediated interactions. However, these structural constraints reduce conformational flexibility and shift some low-frequency vibrational modes toward higher frequencies. Consistently, the average frequency of the five softest modes is higher for the PES minima than for the Gibbs free-energy minima, increasing from 49 to 68 cm^–1^ in geraniol, from 49 to 77 cm^–1^ in nerol, and from 68 to 91 cm^–1^ in linalool. Similar relationships between conformational restriction, low-frequency vibrational modes, and entropy-related effects have been reported in previous MS studies.^50^

Thus, within the MS-HO/RRHO framework, the lower-frequency modes of the extended Gibbs free-energy minima can compensate moderate electronic-energy penalties and make these conformers thermodynamically competitive at 310 K. We highlight that a smaller rotational contribution also favors stabilization. The relative magnitudes of the electronic, rotational, and vibrational contributions are quantified in Section 3.4.

#### 3.3.2 Robustness with Respect to Torsional Anharmonicity

Because the interpretation above relies partly on low-frequency vibrational modes, for which the harmonic approximation is least reliable, the thermodynamic analysis was repeated, including torsional anharmonicity through the MS-T­(C) approximation.

The compact-to-extended shift is preserved in the three systems. For nerol and linalool, the conformer minimizing *G*(310) is the same as in the MS-HO treatment. For geraniol, a different structure formally becomes the Gibbs free-energy minimum, but the interchange is marginal: the three most stable conformers lie within 0.03 kcal mol^–1^ of each other, and all correspond to extended arrangements. In fact, the new MS-T­(C) minimum is even more extended than the MS-HO minimum, with *R*_g_ increasing from 3.05 to 3.17 Å and *d*_O–C_far__ increasing from 8.78 to 9.45 Å.

The populations of the conformers minimizing the Gibbs free energy in the MS-HO and MS-T­(C) treatments are very similar, with differences not exceeding 2.3 percentage points across the three systems. Moreover, the MS-T­(C) and MS-HO conformer rankings are almost identical (ρ_s_ ≥ 0.993 for the three terpenoids), and the association between molecular extension and thermodynamic stabilization is preserved. A detailed comparison is provided in the SI.

Thus, torsional anharmonicity modifies individual relative free energies without altering the overall thermodynamic organization of the ensembles, showing that the compact-to-extended shift is not an artifact of the harmonic treatment of the softest modes.

#### 3.3.3 Sensitivity to the Electronic-Structure Description

The compact–extended balance was further examined by recomputing the relative electronic energies at the ωB97X-D/def2-TZVP and DLPNO-CCSD­(T)/def2-TZVP levels within the MS-HO treatment. Both levels preferentially stabilize compact conformers relative to more extended structures, but the resulting identity of the Gibbs free-energy minimum is system-dependent. The compact-to-extended shift remains robust for geraniol, whereas nerol and linalool show a stronger sensitivity to the electronic-structure description. These results indicate that the detailed thermodynamic ordering depends on the level of theory, although thermal contributions remain sufficiently important to reorganize the underlying electronic-energy landscape. A detailed comparison is provided in the SI.

### 3.4 Global-Shape Descriptors and Thermodynamic Stabilization

To examine whether the thermodynamic stabilization of more extended conformers is a general feature of the conformational landscapes, the relative Gibbs free energies of the conformers were analyzed as a function of the geometric descriptors defined in Table 1.

This comparison showed that the most consistent relationships with *G*_rel_(310) are obtained for global-shape descriptors associated with molecular extension/compactness, whereas descriptors describing local hydroxyl-mediated contacts display more system-dependent trends. Among the descriptors considered, the heavy-atom radius of gyration, *R*_g_, provides the most transferable description across the three terpenoids, indicating that thermodynamic stabilization is more closely related to the overall extension–compactness of the heavy-atom framework than to any single terminal distance. This is particularly relevant for linalool, where branching partly decouples the *d*_O–C_far__ coordinate from the global molecular shape. The complete set of descriptor–energy correlation plots is provided in the SI.

Across the three systems, larger *R*_g_ values are generally associated with lower relative Gibbs free energies (Figure 4), indicating that, globally, more extended conformers tend to be thermodynamically stabilized. Linear regressions give *R* ^2^ values of 0.299, 0.127, and 0.285 for geraniol, nerol, and linalool, respectively. These values indicate that *R*_g_ accounts for a moderate fraction of the thermodynamic variability in geraniol and linalool but a smaller fraction in nerol. The corresponding regression *p*-values (*p*_lin_ = 6.2 × 10^–6^, 1.7 × 10^–2^, and 2.0 × 10^–7^, respectively) are all below the conventional 0.05 threshold, supporting statistically significant linear trends in all three systems. Spearman’s rank correlation further supports this tendency from a nonparametric perspective. Negative Spearman coefficients were obtained for geraniol (ρ_s_ = −0.66), nerol (ρ_s_ = −0.34), and linalool (ρ_s_ = −0.57), showing that larger *R*_g_ values are monotonically associated with lower *G*_rel_(310) values. The association is moderate to strong for geraniol and linalool and weaker for nerol, where the electronic contribution spans a broader energy range and shows little correlation with *R*_g_ (see the SI). Consistently, the corresponding *p*-values (*p*_s_ = 1.2 × 10^–8^, 2.2 × 10^–2^, and 2.5 × 10^–8^, respectively) are all below 0.05, confirming the statistical significance of the monotonic trends.

**Figure 4:**
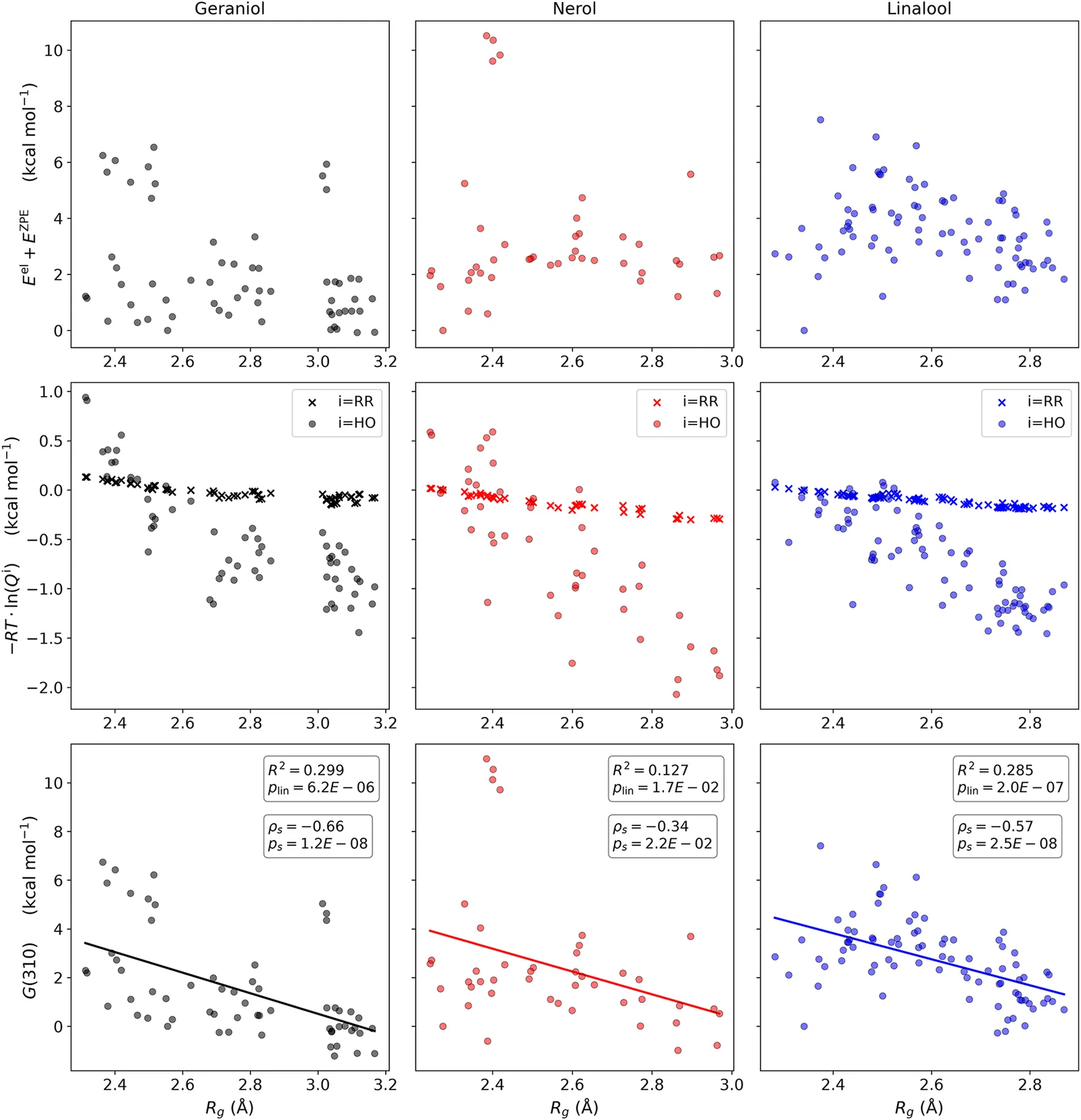
Decomposition of *G*_rel_(310) as a function of *R*_g_ for the conformers of geraniol (left), nerol (center), and linalool (right). Top: Zero-point-inclusive electronic energy; middle: rotational and vibrational contributions to −*RT* ln *Q* ^RRHO^; bottom: resulting *G*_rel_(310). All quantities are referenced to the MPWB1K/6-31+G­(d,p) electronic-energy minimum. Least-squares *R* ^2^ and *p*_lin_ values, together with Spearman’s ρ_s_ and *p*_s_, are reported for *G*_rel_(310) vs *R*_g_.

The physical origin of this relationship can be understood by decomposing *G*_rel_(310) into its electronic and roto-vibrational contributions (eq 2), as shown in Figure 4. The zero-point-inclusive electronic energy (top row) spans the widest range of the three terms, 6.6–10.5 kcal mol^–1^ depending on the system, but shows little correlation with *R*_g_ (*R* ^2^ < 0.11). The rotational contribution (middle row) is the most strongly correlated with molecular extension (*R* ^2^ = 0.79–0.95), as expected from the dependence of the moments of inertia on *R*_g_, yet it spans no more than 0.32 kcal mol^–1^ in any of the three systems. The vibrational contribution also shows a strong correlation (*R* ^2^ = 0.69–0.71) while spanning 1.5–2.7 kcal mol^–1^, roughly 1 order of magnitude more. The trend displayed by *G*_rel_(310) is therefore predominantly thermal and vibrational in origin, whereas the electronic energies account mainly for the residual conformer-to-conformer dispersion. The complete correlation statistics and energy ranges for the individual contributions are reported in the SI.

As shown in Section 3.3.3, the strength of this trend is sensitive to the electronic-structure description. A second factor that may affect this relationship is the surrounding molecular environment. The present results refer to isolated molecules in the gas phase, whereas in condensed or biological environments, intermolecular interactions may compete with the intramolecular contacts that contribute to the compact–extended balance and thereby alter the conformational preferences. The relationship between global extension and thermodynamic stabilization should therefore be regarded as a general but moderate tendency, with *R*_g_ providing the most consistent one-dimensional descriptor among those considered rather than a predictive measure of conformer stability.

### 3.5 Multidimensional Organization of Conformational Ensembles

In this section, the structural variability of the conformational ensembles is examined by using previously defined geometric descriptors. The ranges spanned by these descriptors are reported in the SI, while the descriptor space is analyzed here using PCA and clustering methods to identify the main structural trends that govern the organization of each ensemble.

#### 3.5.1 PCA of the Conformational Ensembles

PCA was performed by using the geometric descriptors previously described. The first three PCs account for most of the total structural variance, explaining 84, 86, and 82% of the variance for geraniol, nerol, and linalool, respectively. This indicates that the structural variability of the conformational ensembles can be described by a limited number of correlated descriptor combinations.

The PCA loading heatmaps are shown in Figure 5. In the three terpenoids, PC1 is dominated by the O–C_far_ separation (D_1_) and by the relative position of the hydroxyl group with respect to the distal unsaturated region (D_5_, D_7_) and can be interpreted primarily as a terminal-extension coordinate, explaining 51, 53, and 47% of the variance in geraniol, nerol, and linalool, respectively. The way the remaining variance is distributed depends on molecular topology.

**Figure 5:**
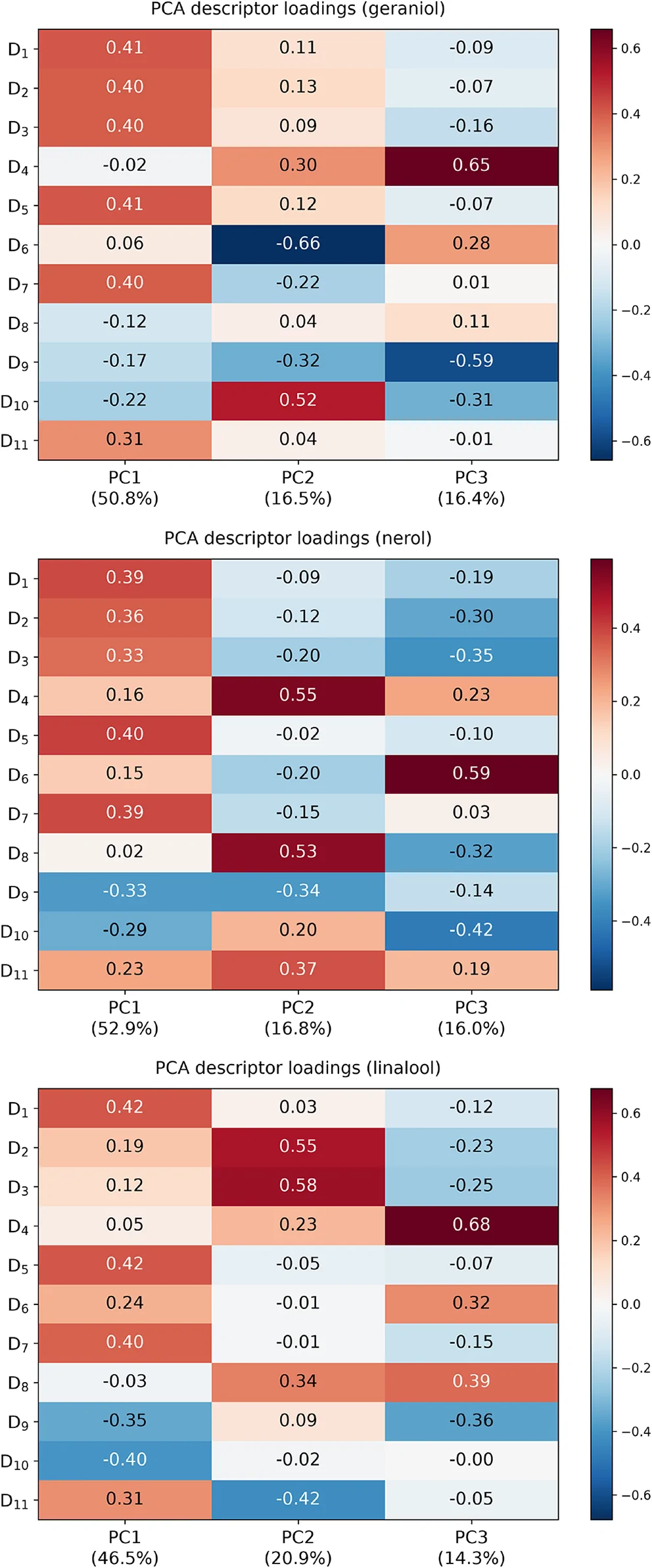
PCA loading heatmaps for geraniol, nerol, and linalool. Descriptor labels D_1_–D_11_ correspond to the definitions given in Table 1; their ranges are reported in the SI. Values indicate the loadings of each descriptor on the first three PCs, with the percentage of explained variance reported below each component.

In the two relatively linear systems, geraniol and nerol, the global-shape descriptors *R*_g_ (D_2_) and κ^2^ (D_3_) load together with PC1, so that terminal extension, radius of gyration, and shape anisotropy vary in a concerted manner, while PC2 and PC3 capture local folding and hydroxyl-mediated contact patterns. In branched linalool, by contrast, *R*_g_ and κ^2^ contribute mainly to PC2, showing that global extension/compactness and shape anisotropy are partly decoupled from the O–C_far_ separation; similar values of one descriptor may then correspond to different global shapes, hydroxyl orientations, or local contact patterns.

This distinction between concerted and decoupled variations is the main structural difference among the PCA representations of the three conformational ensembles.

#### 3.5.2 Clustering and Representative Conformational Regions

*K*-means and GMM analyses were performed in the reduced PCA space to assess whether the conformational ensembles could be partitioned into distinct structural families. None of the validation analyses identified a unique and consistently supported number of conformational regions across the 2–20 group range explored. The *K*-means silhouette and Davies–Bouldin criteria did not show a well-defined optimum, while the GMM BIC profiles depended strongly on the covariance parametrization and differed among the three terpenoids. At larger group numbers, improved validation scores were frequently associated with increasingly small components. The corresponding validation curves, covariance-model comparisons, and reproducibility tests are provided in the SI.

These results indicate that the conformational ensembles are better described as continuous landscapes than as collections of sharply separated structural families. The GMM analysis was therefore used as an interpretable coarse-grained partition of the descriptor space rather than as a model-selection procedure. A four-component representation was retained as an operational compromise among partition stability, population of the resulting regions, and preservation of thermodynamically distinct conformational behavior. At *k* = 3, chemically relevant distinctions between regions are lost, whereas larger partitions introduce very small components and more irregular reproducibility. Importantly, *k* = 4 is the largest common partition for which every component contains more than three structures. This choice does not imply the existence of four discrete physical states.

Using this four-component representation, representative conformational regions were characterized for each terpenoid (Table 4) and ordered by increasing mean radius of gyration, ⟨*R*_g_⟩. Because the Gibbs free-energy distributions may be asymmetric and partially overlapping, their thermodynamic behavior is summarized by the median *G*_rel_(310) and interquartile range (IQR). The mean zero-point-inclusive electronic energy, ⟨*E*_rel_⟩, is also reported to facilitate comparison between electronic and thermodynamic ordering.

**Table 4:** Summary of the Representative Conformational Regions Obtained from the Four-Component GMM Representation^a^

system	region	*N*	⟨*R*_g_⟩	⟨*d*_O–C_far__⟩	⟨*E*_rel_⟩	median *G*_rel_(310)	IQR (*G*_rel_(310))
geraniol	compact I	11	2.47	5.50	1.72	2.32	1.69–3.46
	compact II	11	2.50	5.98	3.87	3.93	2.97–6.94
	extended I	24	2.91	8.28	1.71	1.16	0.91–1.94
	extended II	14	2.97	8.56	2.59	1.91	1.58–2.72
nerol	compact	5	2.34	4.77	1.57	2.35	0.99–2.54
	semicompact	19	2.40	5.55	4.28	3.26	2.82–4.86
	intermediate	10	2.64	6.48	3.32	2.87	2.24–3.82
	extended	10	2.86	8.01	3.23	1.77	1.23–2.70
linalool	folded	9	2.42	4.44	2.72	3.00	2.37–3.39
	folded, terminally extended	11	2.44	6.47	4.12	3.72	3.57–3.83
	extended I	41	2.65	6.42	4.28	3.50	2.18–4.22
	extended II	22	2.68	6.28	2.92	1.55	0.96–2.76

a *N* is the number of conformers assigned to each region. Median relative Gibbs free energies at 310 K and interquartile ranges are reported, with the IQR defined as the range between the first and third quartiles. Energies are given in kcal mol^–1^, and distances are in Å.

The region analysis shows that thermal contributions can substantially reorganize the relative stability of compact and extended regions. In both nerol and linalool, the compact (folded) region has the lowest mean electronic energy, but this ordering is not preserved in Gibbs free energy: the lowest median *G*_rel_(310) is found for an extended region with a higher mean electronic energy. In geraniol, two regions have essentially identical mean electronic energies (1.71 and 1.72 kcal mol^–1^) yet median Gibbs free energies differing by more than 1 kcal mol^–1^, indicating that the relative stabilization of the extended region is predominantly thermal in origin.

This trend is not strictly monotonic in *R*_g_. In linalool, in particular, the two extended regions have very similar mean *R*_g_ values (2.65 and 2.68 Å) but differ by nearly 2 kcal mol^–1^ in median free energy. Moreover, the least thermodynamically favorable region (folded, terminally extended) combines a large mean terminal extension with an *R*_g_ comparable to that of the folded region. Thus, although *R*_g_ provides the clearest one-dimensional descriptor of thermodynamic stabilization across the conformational ensemble, it does not by itself determine the conformational ordering within the branched linalool landscape.

## 4 Conclusions

In this work, the conformational ensembles of geraniol, nerol, and linalool were characterized by combining automated conformational exploration, MS thermodynamics, geometric descriptors, and multivariate analysis. The conformational searches identified numerous accessible minima, but only a subset contributes substantially to the equilibrium ensemble at 310 K; for the three systems, the Gibbs free-energy minima differ from the potential-energy minima. Compact and semifolded structures are favored electronically, whereas more extended conformers become thermodynamically competitive through entropy-related contributions associated with low-frequency vibrational modes; this shift is preserved when torsional anharmonicity is included through the MS-T­(C) approximation, confirming that it is not an artifact of the harmonic treatment. As a consequence, the conformers that dominate the equilibrium distribution can expose hydroxyl orientations, backbone arrangements, and unsaturated moiety environments that differ from those of the global potential-energy minima.

Among the descriptors considered, thermodynamic stabilization is most consistently associated with the radius of gyration, although the association is moderate and its strength depends on the electronic-structure description. A decomposition of the relative Gibbs free energies shows that this trend is predominantly vibrational in origin, with only a minor rotational contribution, and the electronic energies account for most of the residual dispersion among structures of similar extension. Consistent with this, the *K*-means and GMM analyses do not identify sharply separated states: the ensembles are better described as continuous conformational landscapes than as discrete families, with the compact-to-extended shift recovered across entire regions and not only from individual minima. Molecular extension and global shape emerge as the major structural coordinates, although in branched linalool, they are partly decoupled from the terminal-extension coordinate, yielding a more heterogeneous landscape in which similar *R*_g_ values may correspond to different thermodynamic stabilities.

Overall, these results show that single-geometry models do not fully capture the thermodynamic diversity of flexible terpenic alcohols and support the use of ensemble-based representations for flexible odorants and related acyclic terpenoids. It should be stressed, however, that the present calculations describe isolated molecules in the gas phase and include no odorant-receptor modeling: they establish that the thermally dominant conformers differ from the potential-energy minima, but not that this difference changes the outcome of a docking or pharmacophore calculation. Whether ensemble-based representations modify such results and to what extent the compact-versus-extended balance survives in a receptor environment are questions that this work motivates rather than answers and that define the natural continuation of the present study.

## Supplementary Material



## Data Availability

The computational data supporting this work are available at the Zenodo repository: 10.5281/zenodo.20800420. The repository includes the TorsiFlex^18,19^ input and output files used for the conformational exploration of geraniol, nerol, and linalool, together with the files employed for the MsTor^38^ torsional-anharmonicity calculations and the additional ωB97X-D/def2-TZVP and DLPNO-CCSD­(T)/def2-TZVP calculations used to assess the sensitivity of the conformational energetics to the electronic-structure description. These data provide the structural and energetic information underlying the multistructural thermodynamic, descriptor-based, PCA, and clustering analyses reported here.

## Notes

Details of the data repository; TorsiFlex conformational exploration; assessment of torsional anharmonicity through MS-T­(C) partition functions; sensitivity of the compact–extended balance to the electronic-structure description; ranges of the geometric descriptors and the descriptor–Gibbs free-energy correlations not shown in the main text; decomposition of the relative Gibbs free energies into electronic, rotational, and vibrational contributions along the radius of gyration; and clustering-validation and model-selection analyses (PDF)
