# Supplementary material for: Ensemble-Based Descriptor Analysis of Flexible Odorants: Conformational Landscapes of Geraniol, Nerol, and Linalool
Source: J Phys Chem A. 2026 Oct 14;130(43):8557–69. doi: 10.1021/acs.jpca.6c04688 (PMC13726388; doi:10.1021/acs.jpca.6c04688)
Supplement: Supplementary file 1 [file jp6c04688_si_001.pdf]

Supporting Information (SI) for

Ensemble-Based Descriptor Analysis of Flexible Odorants: Conformational Landscapes of Geraniol, Nerol, and Linalool

Beatriz Cuadrado-Benavent[a], Juan J. Nogueira*[a,b], and David Ferro-Costas*[c]

[a]Department of Chemistry, Universidad Autónoma de Madrid, Calle Francisco Tomás y Valiente, 7, 28049 Madrid, Spain.
[b]Institute for Advanced Research in Chemical Sciences (IAdChem), Universidad Autónoma de Madrid, 28049, Madrid, Spain
[c]Departamento de Química Física, Universidade de Santiago de Compostela, 15782 Santiago de Compostela, Spain

Contents

S1 Data and repository contents **S2**
S2 Details of the `TorsiFlex` conformational exploration **S3**
S3 Effect of torsional anharmonicity **S5**
S4 Sensitivity to the electronic-structure description **S6**
S4.1 Changes in relative electronic energies S6
S4.2 Consequences for thermodynamic ordering S8
S5 Geometric descriptor ranges and Gibbs free-energy correlations **S9**
S6 Decomposition of the Gibbs free energy along the extension coordinate **S20**
S7 Determination of the number of conformational families **S24**
S7.1 Assessment of K-means partitions S24
S7.2 Gaussian mixture models and BIC profiles S25
S7.3 Choice of the number of components S27

*Corresponding authors: juan.nogueira@uam.es; david.ferro@usc.es

S1. Data and repository contents

Additional data associated with this work are available in the Zenodo repository:

- `https://doi.org/10.5281/zenodo.20800420`

which is organized into four main folders, each containing separate subfolders for geraniol, nerol, and linalool:

- `1-Search/`: `TorsiFlex` input and output files associated with the conformational exploration and the MPWB1K/6-31+G(d,p) optimized structures.
- `2-MsTor/`: files used for the multistructural torsional-anharmonicity calculations performed with `MsTor`.
- `3-wb97xd/`: data from the ωB97X-D/def2-TZVP calculations used to assess the sensitivity of the conformational energetics to the electronic-structure description.
- `4-ccsdt/`: data from the DLPNO-CCSD(T)/def2-TZVP calculations used as an additional high-level assessment of the conformer energetics.

These files document the conformational search and subsequent electronic-structure and thermodynamic analyses reported in this work, and provide the structural and energetic data underlying the multistructural thermodynamic, descriptor-based, PCA, and clustering analyses discussed in the manuscript.

S2. Details of the TorsiFlex conformational exploration

The active torsional coordinates used for each molecule are defined in Section 2.1 and Fig. 1 of the main manuscript and are therefore not repeated here. This section reports the numerical settings used in the TorsiFlex searches and the criteria used to reject previously sampled regions and duplicate conformers.

The preconditioned stage used the default TorsiFlex values of $60°$, $180°$, and $300°$ for each target torsion, corresponding to the *gauche*$^+$, *anti*, and *gauche*$^-$ arrangements described in the main manuscript. No external preconditioning file or restricted torsional domains were used, so the stochastic stage sampled the full torsional domain of each active coordinate.

The stochastic search was performed in successive batches of 2500 cycles (`ncycles = 2500`). TorsiFlex stores the torsional vectors associated with previously generated and optimized structures and uses them in the similarity test to avoid resampling their local neighborhoods. Successive stochastic searches were therefore continued from the accumulated search history. For each terpenoid, the stochastic exploration was repeated until a complete additional 2500-cycle search produced no new conformer; this condition was used as the practical convergence criterion of the conformational search.

The validation parameters were identical for the three terpenoids and are summarized in Table S1. The connectivity and similarity tests were active for generated trial geometries, and the connectivity and redundancy tests were active after optimization. No hard or soft geometric constraints were imposed. The connectivity graph was constructed with `cfactor = 1.3`. In the similarity test, a generated torsional vector lying within 7° in every active torsion of a previously stored vector was regarded as belonging to an already sampled region and was not reoptimized. After geometry optimization, two structures were regarded as torsionally redundant when the corresponding active torsions agreed within 2°. Structures passing these tests were subjected to a Hessian calculation and retained only when they corresponded to minima.

Table S1: TorsiFlex settings used in the conformational searches.

Setting	Geraniol	Nerol	*(R)*-Linalool
Stochastic cycles (`ncycles`)	2500	2500	2500
Connectivity factor (`cfactor`)	1.3	1.3	1.3
Guess tests (`testsG`)	1 1 1 1	1 1 1 1	1 1 1 1
Optimized tests (`testsO`)	1 1 1 1	1 1 1 1	1 1 1 1
Similarity domain (`dist1D`)	7°	7°	7°
Redundancy threshold (`epsdeg`)	2°	2°	2°
Hard/soft constraints	none	none	none
High-level cutoff (`hlcutoff`)	none	none	none
LL Hessian in HL opt. (`fccards`)	no	no	no
Torsional enantiomerism (`enantio`)	yes	yes	no

Torsional enantiomerism was enabled for geraniol and nerol (`enantio = yes`), allowing `TorsiFlex` to exploit the mirror-related torsional vectors during the search, whereas it was disabled for the explicitly studied *(R)*-linalool enantiomer (`enantio = no`). No high-level energy cutoff was applied, so all distinct low-level conformers retained after the HF/3-21G exploration were submitted to high-level reoptimization and validation.

Figure S1 shows the distribution of relative electronic energy for all conformers retained after the conformational exploration and high-level refinement. Each vertical mark corresponds to one conformer, providing a compact representation of the energetic spread of the final conformational ensembles for geraniol, nerol, and linalool.

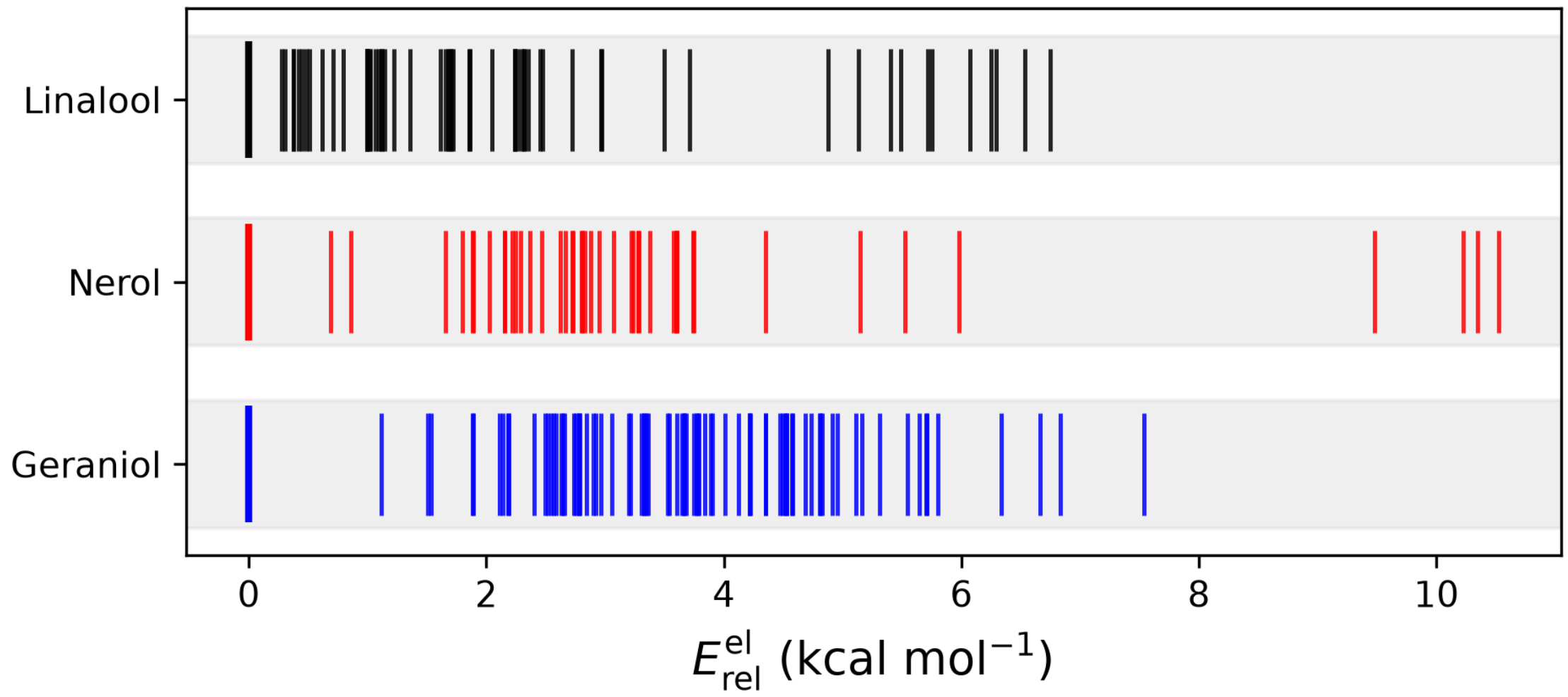

Figure S1: Distribution of relative total electronic energies, E_{rel}^{el}, for the conformers retained for geraniol, nerol, and linalool. Each vertical mark represents one conformer in the final conformational ensemble.

S3. Effect of torsional anharmonicity

The multistructural harmonic-oscillator (MS-HO) treatment employed throughout the manuscript describes all vibrational modes, including the softest torsional ones, within the harmonic approximation. To assess the reliability of this description, the partition functions were re-evaluated within the multistructural torsional approximation with coupled potential, MS-T(C), as implemented in the `MSTor` program, which replaces the harmonic treatment of the torsional modes by a hindered-rotor description including couplings among the torsions and between the torsions and the remaining degrees of freedom.

Figure S2 compares the relative Gibbs free energies at 310 K obtained with both approximations for all conformers of the three terpenoids, using as a common reference the conformer that minimizes the electronic energy. The points follow the diagonal closely over the whole energy range, showing that torsional anharmonicity shifts the relative Gibbs free energies by comparatively small amounts and does not reorganize the thermodynamic hierarchy of the ensembles. Consistently, the Spearman rank correlation coefficients between the MS-T(C) and MS-HO orderings are 0.993, 0.995 and 0.997 for geraniol, nerol and linalool, respectively.

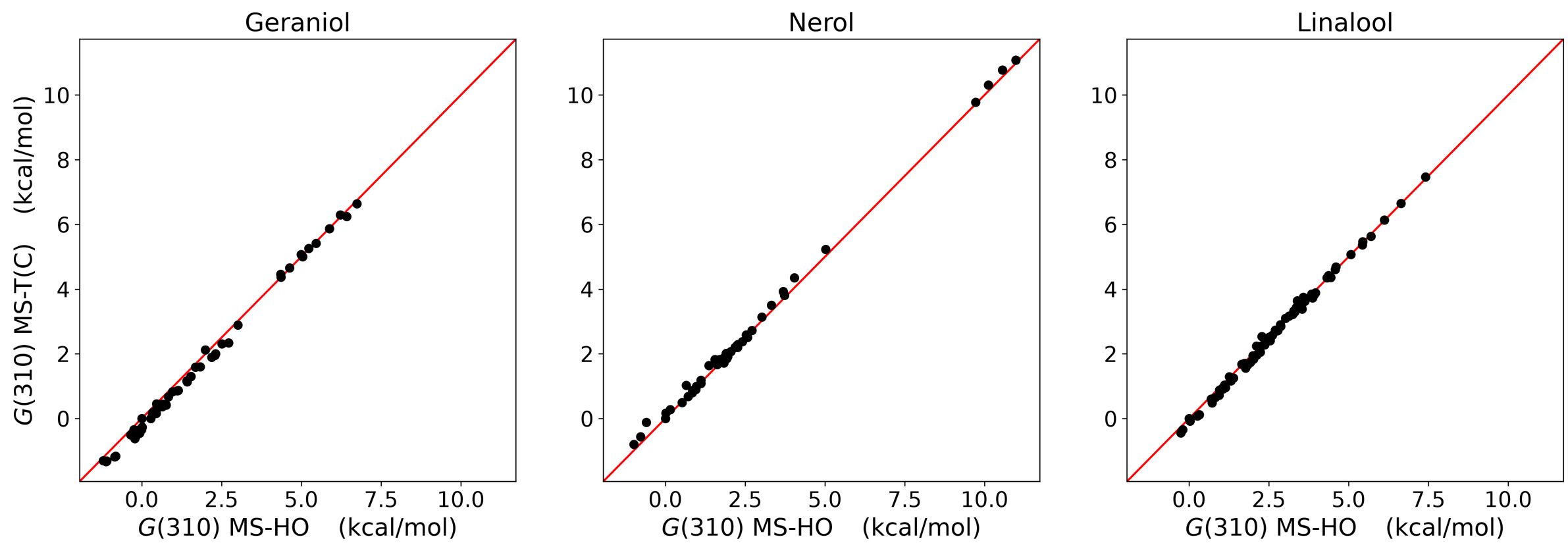

Figure S2: Relative Gibbs free energies at 310 K obtained within the MS-T(C) approximation as a function of the corresponding MS-HO values for all conformers of geraniol (left), nerol (center) and linalool (right). Both quantities are referred to a common reference conformer, the one minimizing the electronic energy, so that deviations from the diagonal ($y = x$) measure the effect of torsional anharmonicity on the relative stability of each structure.

The conformers minimizing $G(310)$ within both approximations were also compared. For nerol and linalool the Gibbs free-energy minimum is the same in the two treatments. For geraniol a different structure becomes the minimum under MS-T(C), but the three most stable conformers lie within 0.03 kcal mol^{-1} of each other, all of them correspond to extended arrangements, and the population of the MS-HO minimum decreases only from 13.8% to 11.1%. The interchange therefore reflects a local reordering among nearly degenerate structures rather than a change in the compact-to-extended picture discussed in the main manuscript.

S4. Sensitivity to the electronic-structure description

To assess the sensitivity of the compact–extended energy balance to the underlying electronic-structure treatment, single-point electronic energies were recomputed on the MPWB1K/6-31+G(d,p) geometries at the ωB97X-D/def2-TZVP and DLPNO-CCSD(T)/def2-TZVP levels, the latter providing a coupled-cluster reference. No geometry reoptimization or frequency calculation was performed at these alternative levels.

S4.1. Changes in relative electronic energies

We first examined how the relative electronic energies change with respect to the MPWB1K description. To ensure a direct comparison between methods, all relative electronic energies were referred to the same structure, namely the conformer minimizing the electronic energy at the MPWB1K/6-31+G(d,p) level. For each conformer i and electronic-structure method M, the change with respect to MPWB1K was quantified as

$$\Delta\Delta E_i^{\mathrm{el},M} = E_{\mathrm{rel},i}^{\mathrm{el},M} - E_{\mathrm{rel},i}^{\mathrm{el,MPWB1K}}, \qquad \text{(S1)}$$

where, for every method M, $E_{\mathrm{rel},i}^{\mathrm{el},M} = E_i^{\mathrm{el},M} - E_{\mathrm{ref}}^{\mathrm{el},M}$, with the reference conformer being the MPWB1K electronic-energy minimum.

Figure S3 shows $\Delta\Delta E^{\mathrm{el}}$ as a function of R_g for the three terpenoids and the two alternative electronic-structure levels. Both alternative treatments produce the same qualitative tendency: $\Delta\Delta E^{\mathrm{el}}$ increases with R_g in the three systems, indicating preferential stabilization of compact conformers relative to more extended structures. The effect is more pronounced at the ωB97X-D level, but remains evident with DLPNO-CCSD(T). Thus, the compact–extended electronic-energy balance is sensitive to the treatment of dispersion and electron correlation, although the magnitude of the effect depends on the method and on the molecular system.

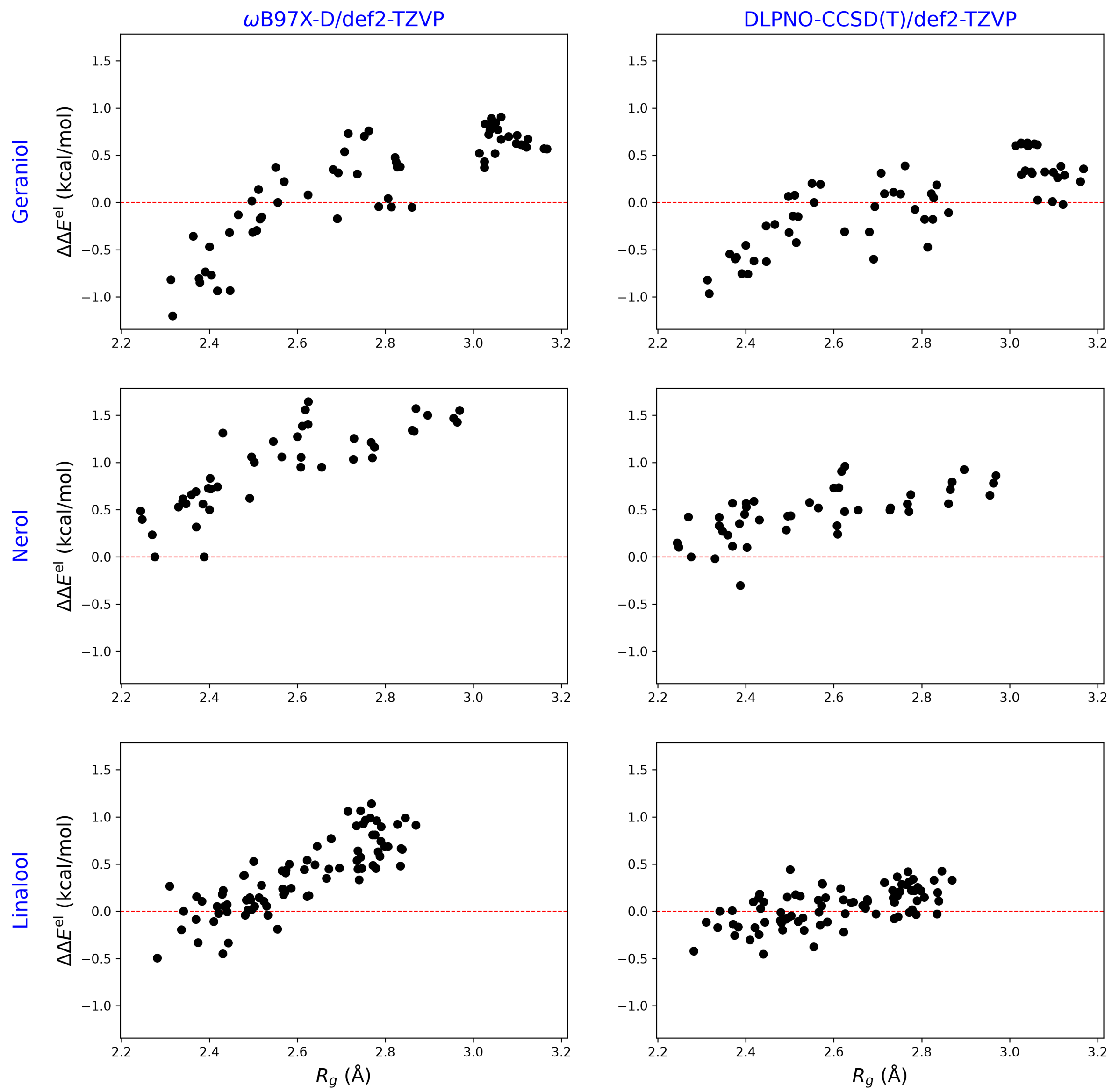

Figure S3: Change in relative electronic energy, $\Delta\Delta E^{\mathrm{el}}$, upon replacing the MPWB1K/6-31+G(d,p) electronic energies by ωB97X-D/def2-TZVP (left column) or DLPNO-CCSD(T)/def2-TZVP (right column) single-point energies, shown as a function of the heavy-atom radius of gyration, R_g, for geraniol (top), nerol (middle), and linalool (bottom).

S4.2. Consequences for thermodynamic ordering

To determine how these changes in electronic energy affect the thermodynamic ordering, Gibbs free energies at the alternative electronic-structure levels were estimated by replacing only the electronic-energy contribution while retaining the zero-point, rotational, and vibrational terms obtained at MPWB1K/6-31+G(d,p). For each conformer i and method M,

$$G_i^M(310) = E_i^{\text{el,M}} + \left[G_i^{\text{MPWB1K}}(310) - E_i^{\text{el,MPWB1K}}\right] \tag{S2}$$

Table S2 summarizes the electronic- and Gibbs free-energy minima and the corresponding $\Delta G(E_{\text{min}})$ between them.

Table S2: Electronic- and Gibbs free-energy minima obtained with the different electronic-structure treatments. R_g values are given in Å and $\Delta G(E_{\text{min}})$ in kcal mol^{-1}.

System	Level	$R_g(E_{\text{min}})$	$R_g(G_{\text{min}})$	$\Delta G(E_{\text{min}})$
Geraniol	MPWB1K	2.56	3.05	1.21
	ωB97X-D	2.38	3.05	0.67
	DLPNO-CCSD(T)	2.38	3.05	1.14
Nerol	MPWB1K	2.28	2.86	0.99
	ωB97X-D	2.28	2.39	0.60
	DLPNO-CCSD(T)	2.28	2.39	0.90
Linalool	MPWB1K	2.34	2.73	0.26
	ωB97X-D	2.34	2.34	0.00
	DLPNO-CCSD(T)	2.34	2.73	0.04

For geraniol, both alternative levels select the same new electronic-energy minimum ($R_g = 2.38$ Å), whereas the Gibbs free-energy minimum remains the extended conformer obtained with MPWB1K ($R_g = 3.05$ Å). The compact-to-extended thermodynamic shift is therefore preserved. The new electronic-energy minimum lies 0.67 and 1.14 kcal mol^{-1} above the Gibbs free-energy minimum at the ωB97X-D and DLPNO-CCSD(T) levels, respectively.

For nerol, the electronic-energy minimum remains unchanged ($R_g = 2.28$ Å), while both alternative treatments select the same new Gibbs free-energy minimum ($R_g = 2.39$ Å), replacing the considerably more extended MPWB1K minimum ($R_g = 2.86$ Å). Thermal contributions therefore continue to reorder the electronic-energy landscape, but the resulting thermodynamic minimum is substantially more compact. The original MPWB1K Gibbs free-energy minimum lies 0.94 and 0.63 kcal mol^{-1} above the new minimum at the ωB97X-D and DLPNO-CCSD(T) levels, respectively.

Linalool displays the strongest sensitivity to the electronic-structure description. Its electronic-energy minimum ($R_g = 2.34$ Å) is unchanged at both alternative levels. At the ωB97X-D level this same conformer also becomes the Gibbs free-energy minimum, whereas at the DLPNO-CCSD(T) level the Gibbs free-energy minimum remains the more extended conformer with $R_g = 2.73$ Å. The two structures are, however, nearly degenerate at the DLPNO-CCSD(T) level, with the compact electronic-energy minimum only 0.04 kcal mol^{-1} above the Gibbs free-energy minimum. The identity of the thermodynamic minimum of linalool should therefore be regarded as particularly sensitive to the electronic-structure treatment.

Overall, the preferential stabilization of compact conformers is common to the two alternative treatments, but its impact on the thermodynamic ordering is system dependent. The compact-to-extended shift is robust for geraniol, substantially reduced for nerol, and method dependent for linalool.

S5. Geometric descriptor ranges and Gibbs free-energy correlations

The ranges spanned by the geometric descriptors used to characterize the conformational ensembles are summarized in Table S3. These values provide the numerical context for the descriptor–energy correlations and multivariate analyses discussed below.

Table S3: Ranges of the geometric descriptors used to characterize the conformational ensembles of geraniol, nerol, and linalool. Distances are given in Å, angles in degrees, and dimensionless descriptors are reported without units.

**Descriptor**		**Geraniol**	**Nerol**	**Linalool**
**(D_1)**	$d_{\mathrm{O-C_{far}}}$	4.20–9.45	4.44–8.39	4.18–7.13
**(D_2)**	R_g	2.31–3.17	2.24–2.97	2.28–2.87
**(D_3)**	κ^2	0.15–0.79	0.12–0.59	0.27–0.66
**(D_4)**	$d_{\mathrm{O}\cdots\pi,1}$	2.63–2.97	2.60–2.97	2.42–3.00
**(D_5)**	$d_{\mathrm{O}\cdots\pi,2}$	4.23–7.70	3.23–6.68	3.13–5.40
**(D_6)**	$d_{\mathrm{H_O}\cdots\pi,1}$	2.67–3.78	2.31–3.77	2.25–3.76
**(D_7)**	$d_{\mathrm{H_O}\cdots\pi,2}$	3.69–8.51	2.64–7.03	2.53–6.17
**(D_8)**	$d_{\mathrm{O\cdots H_C,min}}$	1.99–2.06	1.99–2.06	2.29–2.54
**(D_9)**	c_{O}	6.04 – 8.45	6.08 – 9.55	9.45 – 12.30
**(D_{10})**	$c_{\mathrm{H_O}}$	4.56 – 8.48	4.58 – 12.47	7.22 – 13.48
**(D_{11})**	$\theta_{\mathrm{O-C_\alpha-C_{far}}}$	51–158	50–165	48–171

The relationship between $G_{\mathrm{rel}}(310)$ and R_g is already presented and discussed in the main manuscript. Figures S4–S13 show the corresponding one-dimensional correlations for the remaining geometric descriptors. For each descriptor, the coefficient of determination (R^2), the linear-regression p-value (p_{lin}), the Spearman rank coefficient (ρ_s), and its p-value (p_s) are reported. Consistent with the main text, the most transferable relationships are obtained for global-shape descriptors, whereas local hydroxyl-interaction descriptors show more system-dependent trends.

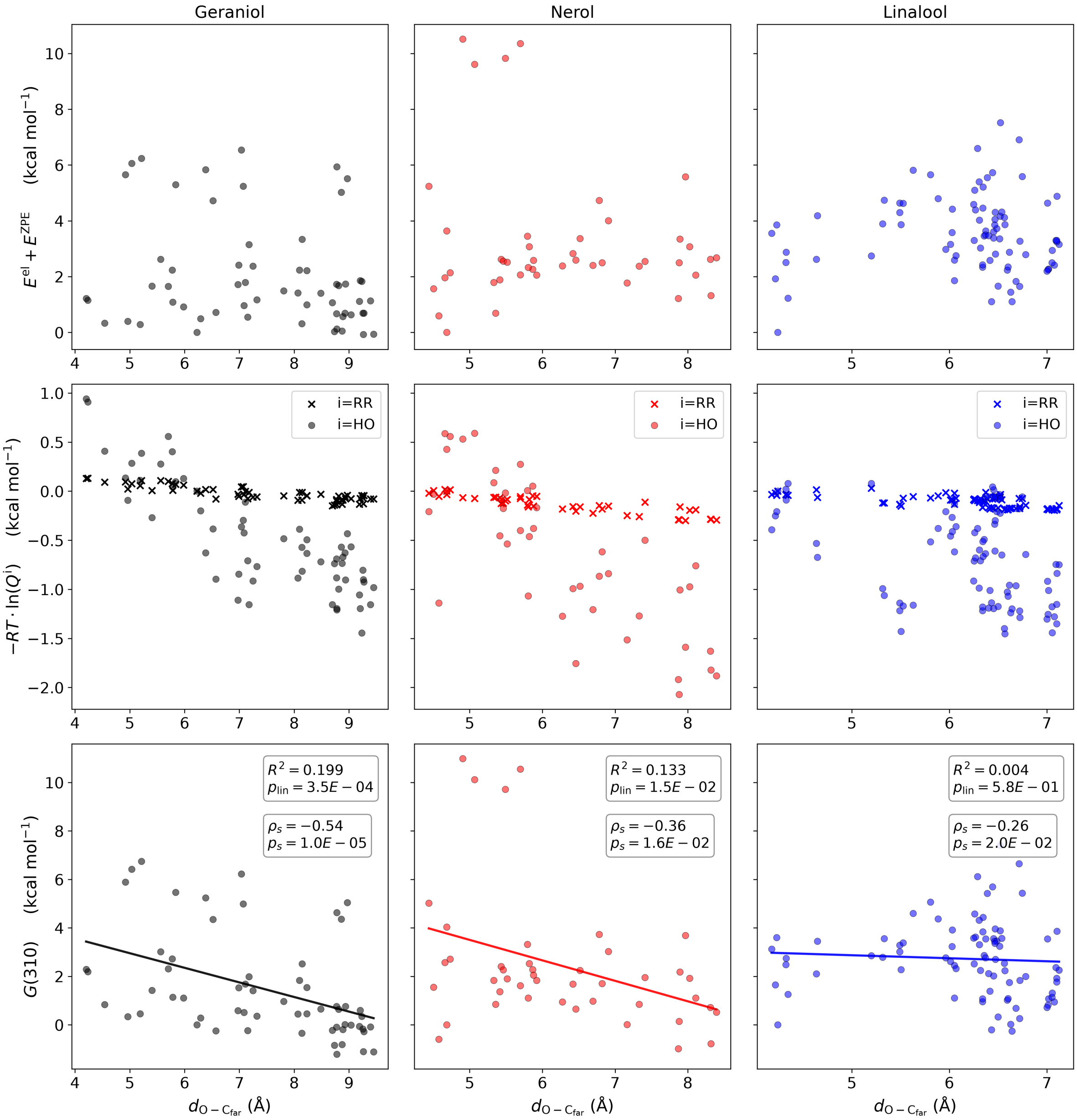

Figure S4: Decomposition of $G_{\text{rel}}(310)$ as a function of $d_{\text{O}-\text{C}_{\text{far}}}$ for the conformers of geraniol (left), nerol (center), and linalool (right). Top: zero-point-inclusive electronic energy; middle: rotational and vibrational contributions to $-RT\ln Q^{\text{RRHO}}$; bottom: resulting $G_{\text{rel}}(310)$. All quantities are referenced to the MPWB1K/6-31+G(d,p) electronic-energy minimum. Least-squares R^2 and p_{lin} values, together with Spearman ρ_s and p_s, are reported for $G_{\text{rel}}(310)$ versus $d_{\text{O}-\text{C}_{\text{far}}}$.

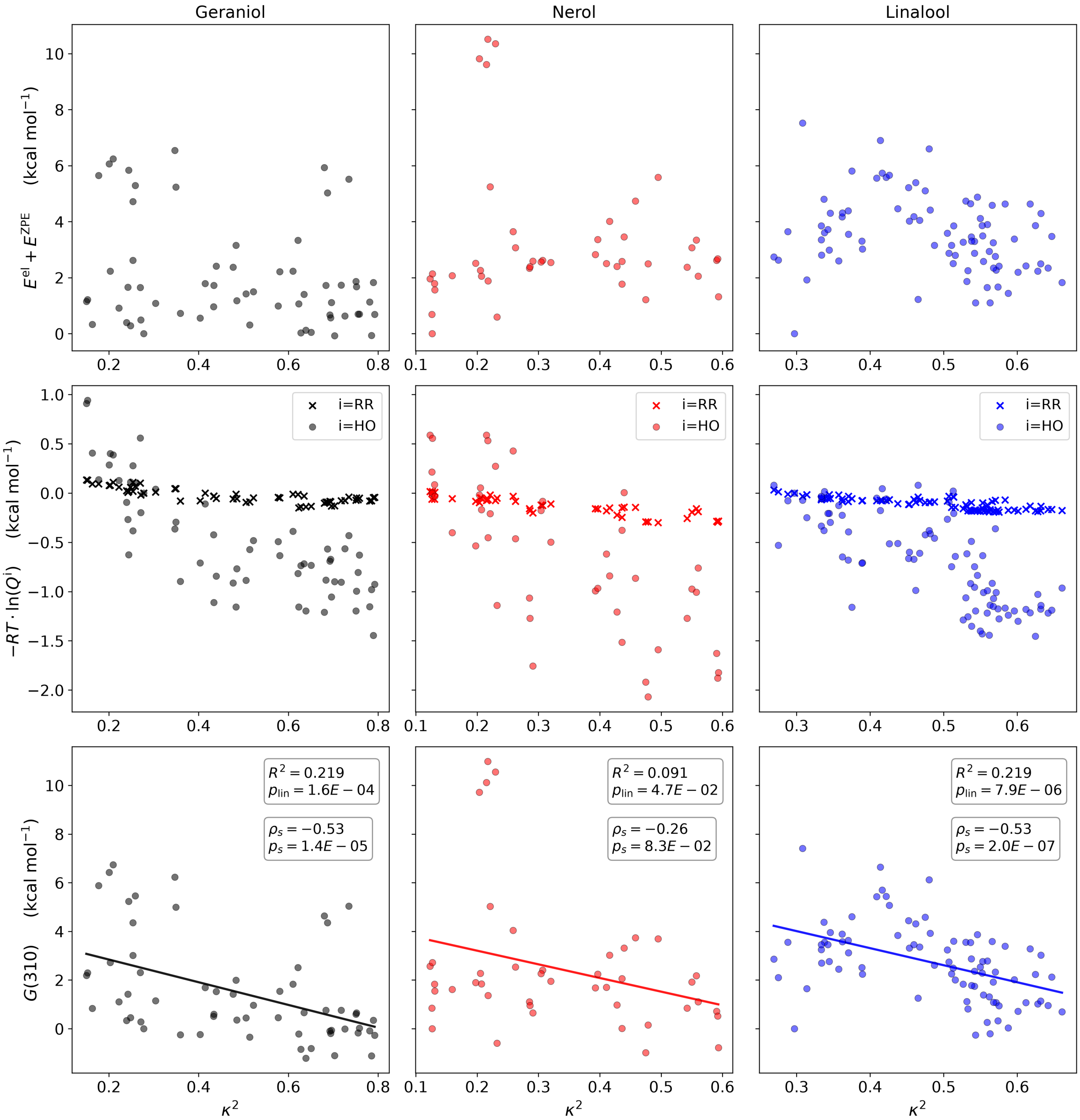

Figure S5: As in Fig. S4, for κ^2.

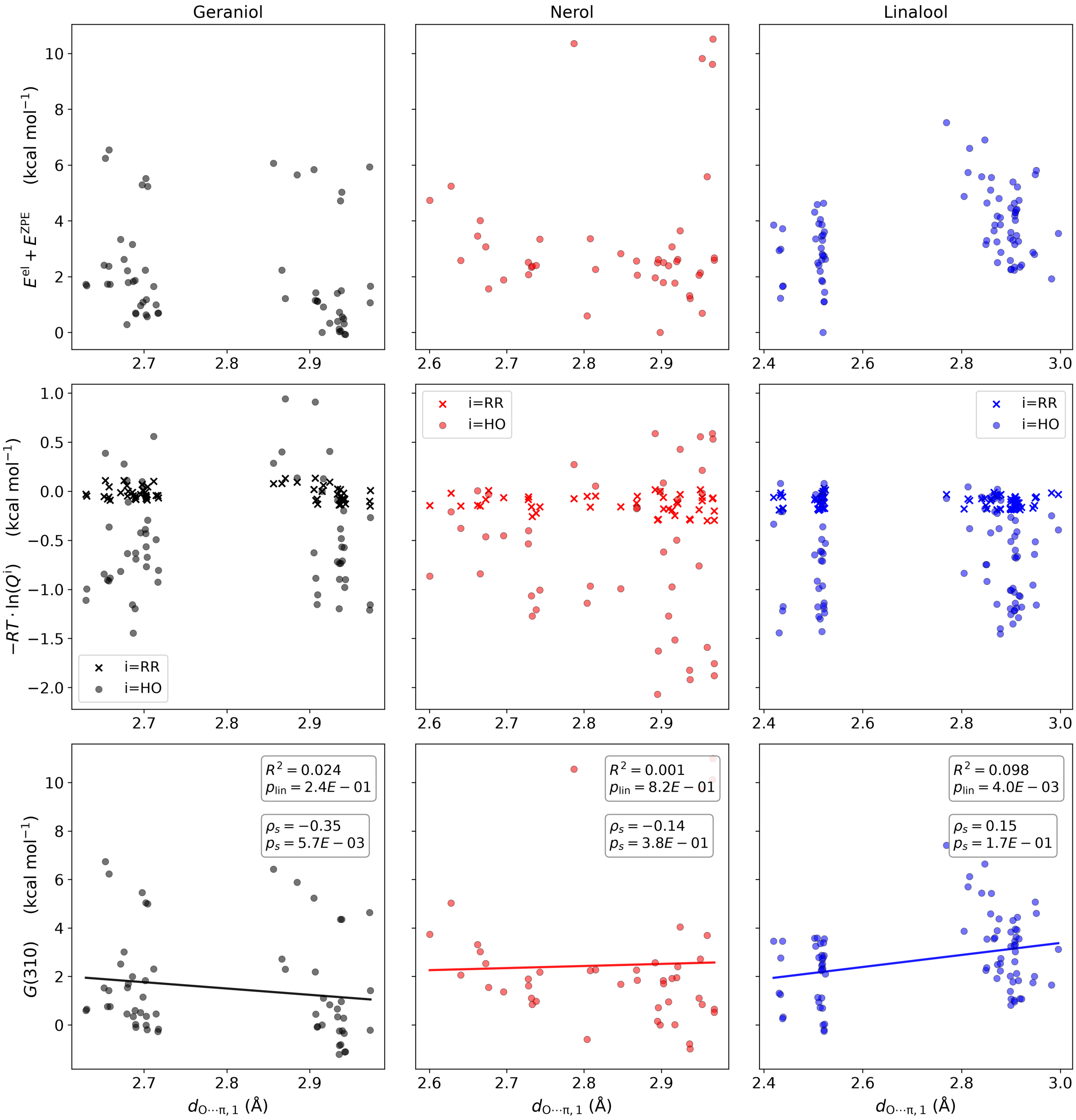

Figure S6: As in Fig. S4, for $d_{O\cdots\pi,1}$.

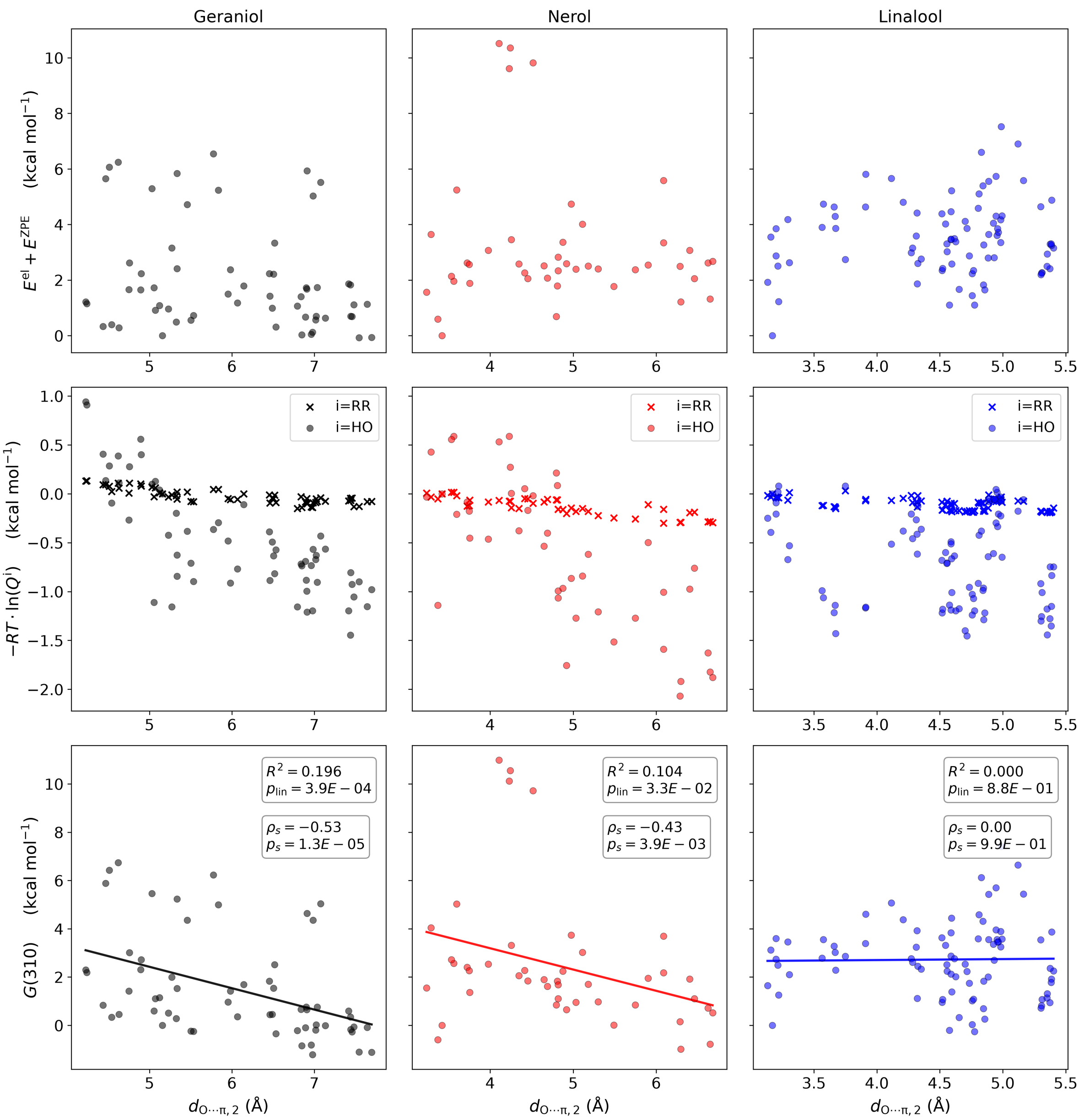

Figure S7: As in Fig. S4, for $d_{O\cdots\pi,2}$.

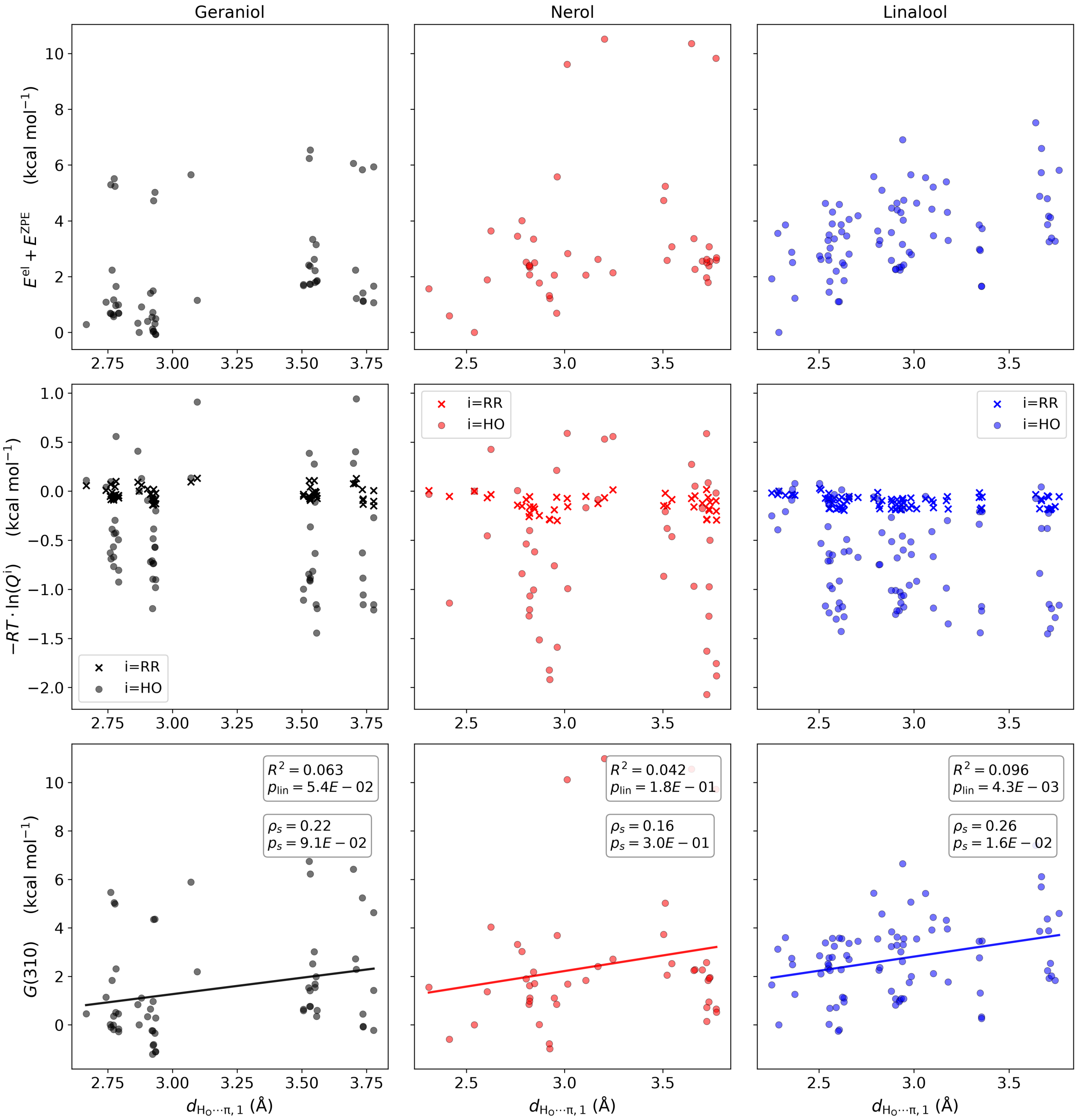

Figure S8: As in Fig. S4, for $d_{H_O \cdots \pi,1}$.

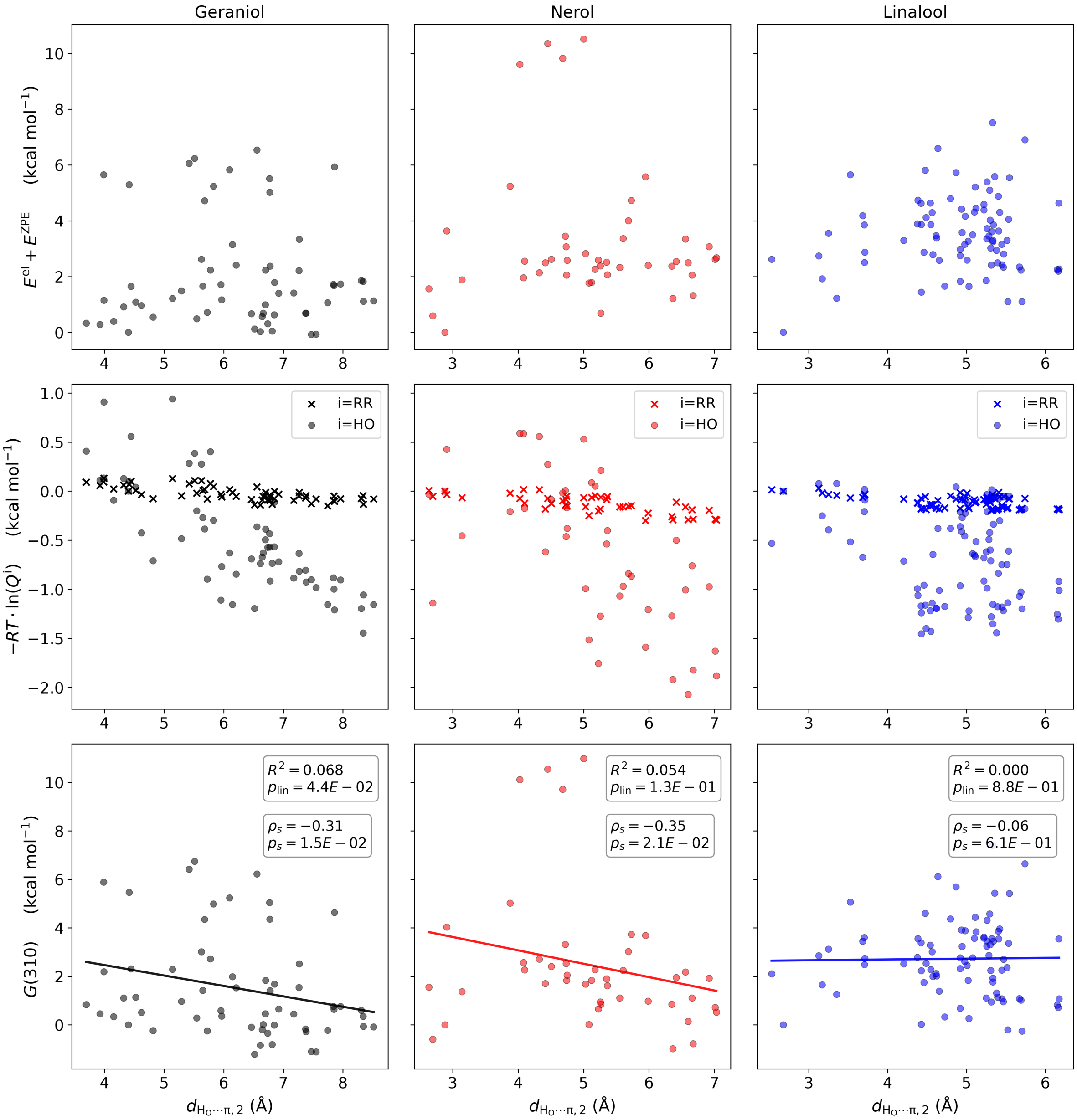

Figure S9: As in Fig. S4, for $d_{H_O \cdots \pi,2}$.

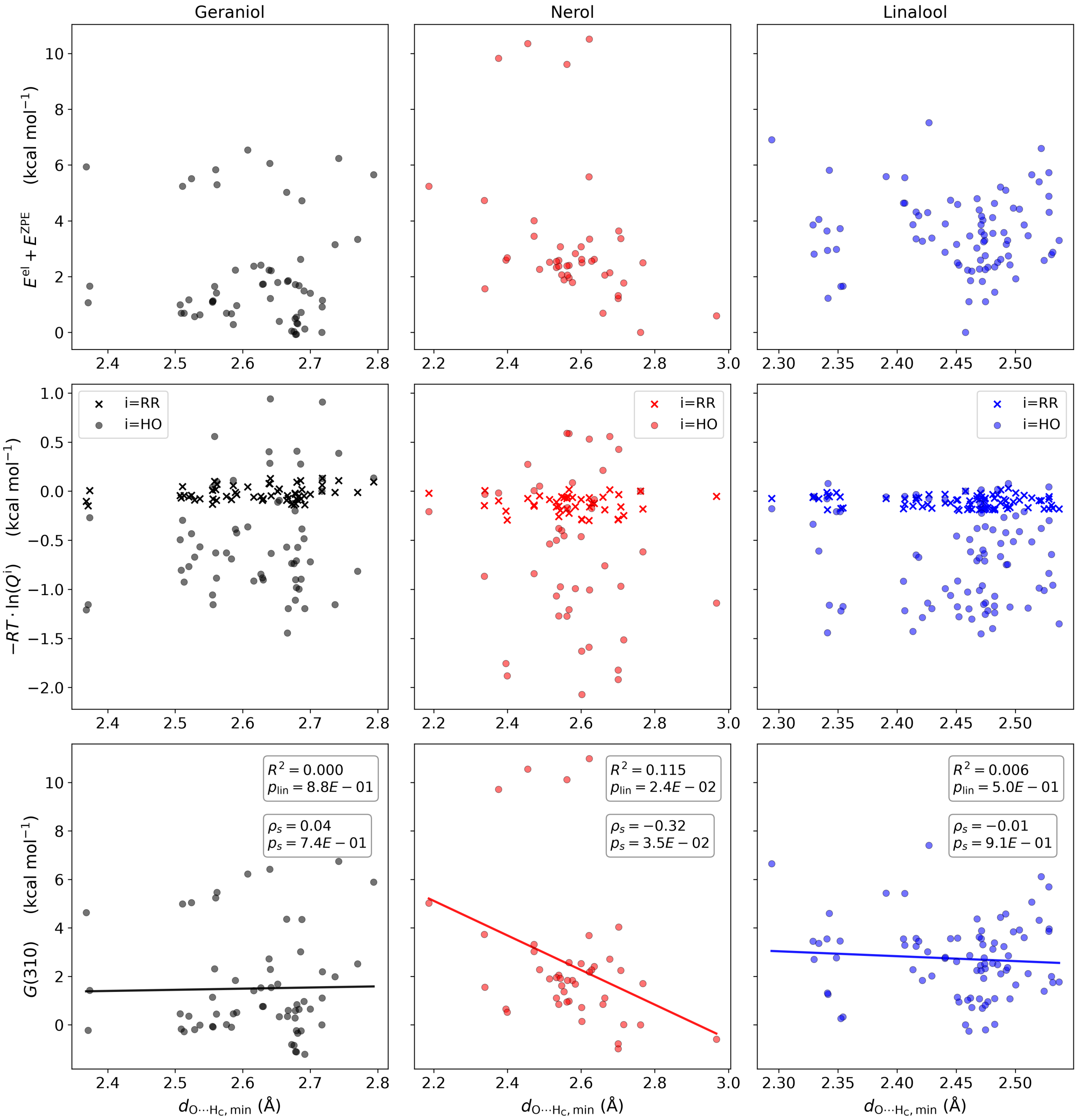

Figure S10: As in Fig. S4, for $d_{O\cdots H_C,\min}$.

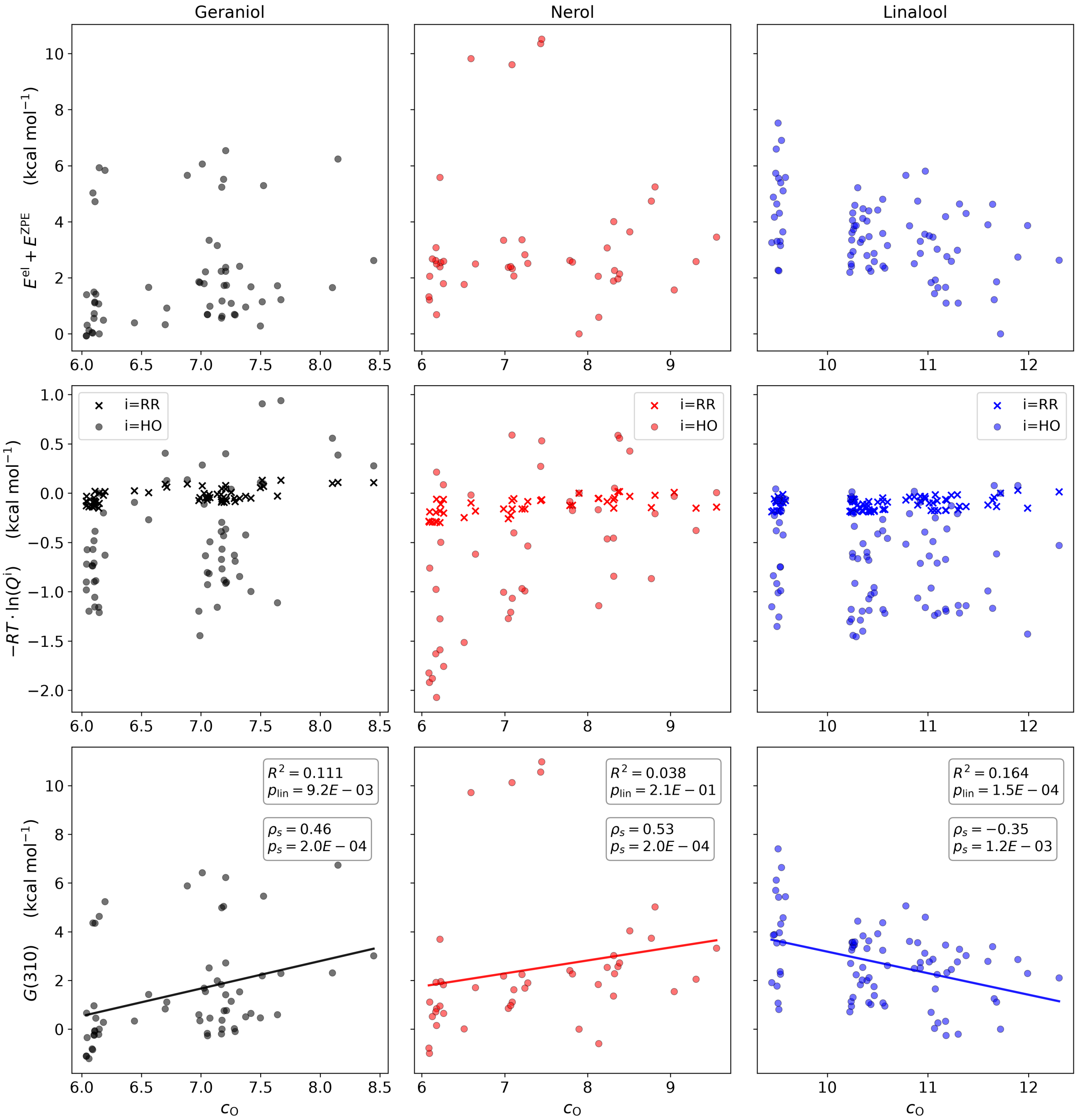

Figure S11: As in Fig. S4, for c_O.

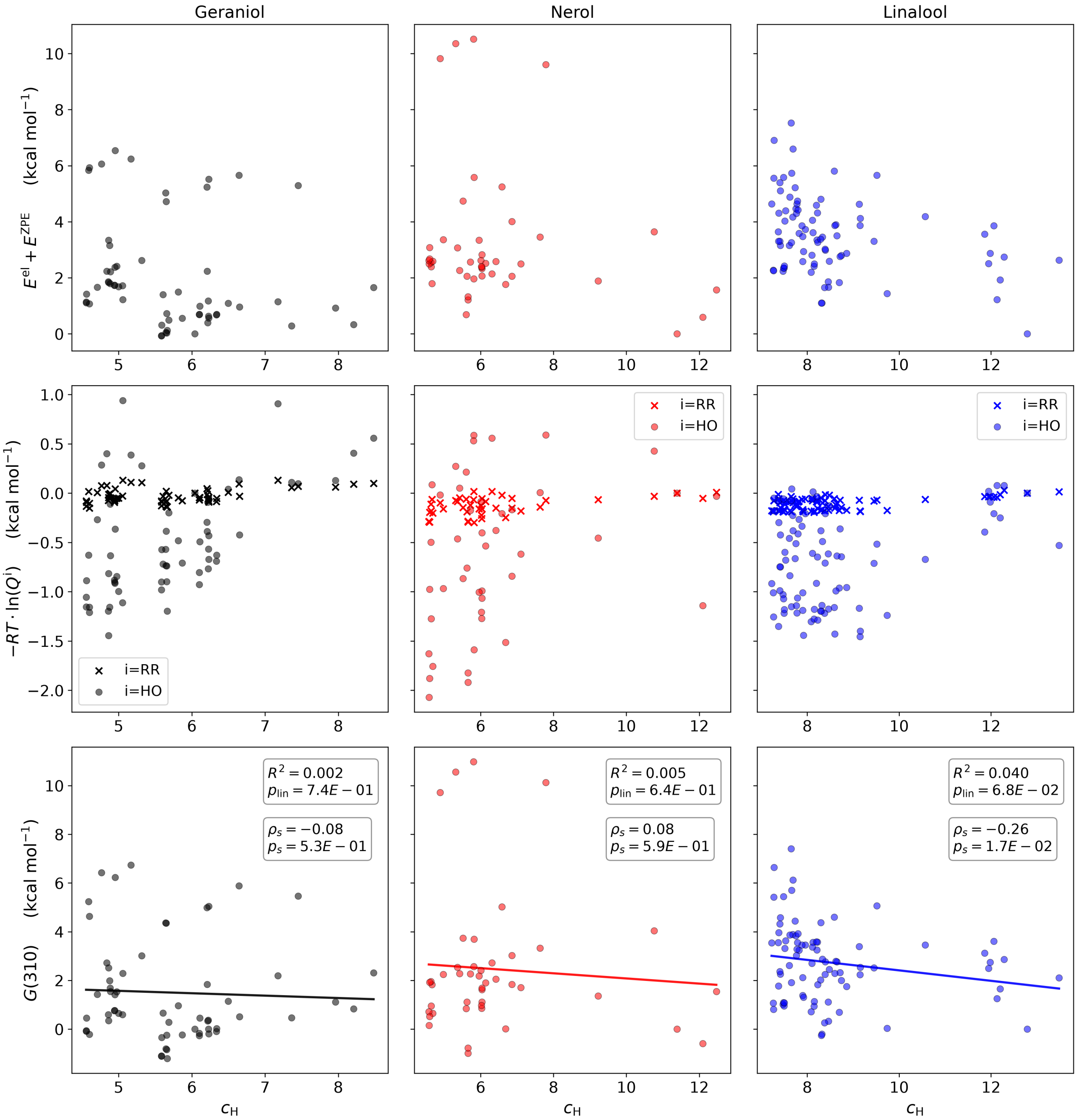

Figure S12: As in Fig. S4, for c_{H_O}.

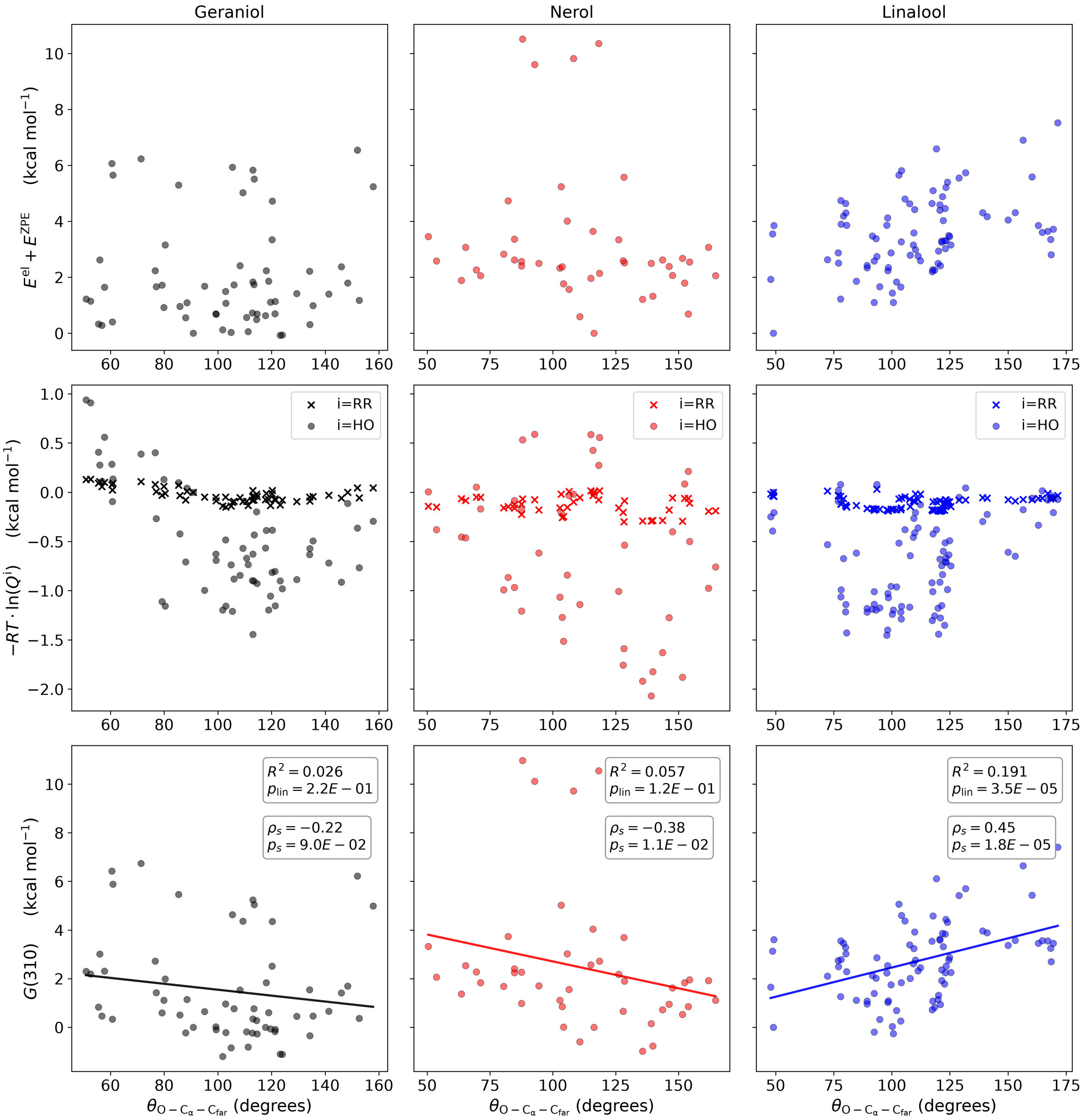

Figure S13: As in Fig. S4, for $\theta_{O-C_\alpha-C_{far}}$.

S6. Decomposition of the Gibbs free energy along the extension coordinate

The association between global molecular extension and thermodynamic stabilization discussed in Section 3.4 of the main manuscript results from the combination of several contributions to the Gibbs free energy. Within the one-well expression of Eq. (1), each conformer contributes through its zero-point-inclusive electronic energy, $E^{\mathrm{el}} + E^{\mathrm{ZPE}}$, and through the rotational and vibrational components of the RRHO partition function,

$$G = \left(E^{\mathrm{el}} + E^{\mathrm{ZPE}}\right) - RT \ln Q_{\mathrm{rot}}^{\mathrm{RR}} - RT \ln Q_{\mathrm{vib}}^{\mathrm{HO}} \tag{S3}$$

Table S4 summarizes the relationship between each contribution and R_g for the conformers retained in the descriptor-based statistical analyses. Figures S14–S16 show the individual contributions as a function of R_g for the three terpenoids.

Table S4: Correlation between R_g and $G_{\mathrm{rel}}(310)$. For each contribution, the energy range (kcal mol^{-1}), the coefficient of determination of the linear regression (R^2), and the Spearman rank coefficient (ρ_s) are reported.

**System**	**Contribution**	**Range**	R^2	ρ_{s}
Geraniol	$E^{\mathrm{el}} + E^{\mathrm{ZPE}}$	6.62	0.109	-0.35
	$-RT \ln Q_{\mathrm{rot}}$	0.28	0.786	-0.84
	$-RT \ln Q_{\mathrm{vib}}$	2.38	0.688	-0.81
	$G_{\mathrm{rel}}(310)$	7.95	0.299	-0.66
Nerol	$E^{\mathrm{el}} + E^{\mathrm{ZPE}}$	10.51	0.013	+0.18
	$-RT \ln Q_{\mathrm{rot}}$	0.32	0.949	-0.96
	$-RT \ln Q_{\mathrm{vib}}$	2.66	0.712	-0.81
	$G_{\mathrm{rel}}(310)$	11.97	0.127	-0.34
Linalool	$E^{\mathrm{el}} + E^{\mathrm{ZPE}}$	7.52	0.083	-0.32
	$-RT \ln Q_{\mathrm{rot}}$	0.22	0.909	-0.92
	$-RT \ln Q_{\mathrm{vib}}$	1.53	0.690	-0.81
	$G_{\mathrm{rel}}(310)$	7.67	0.285	-0.57

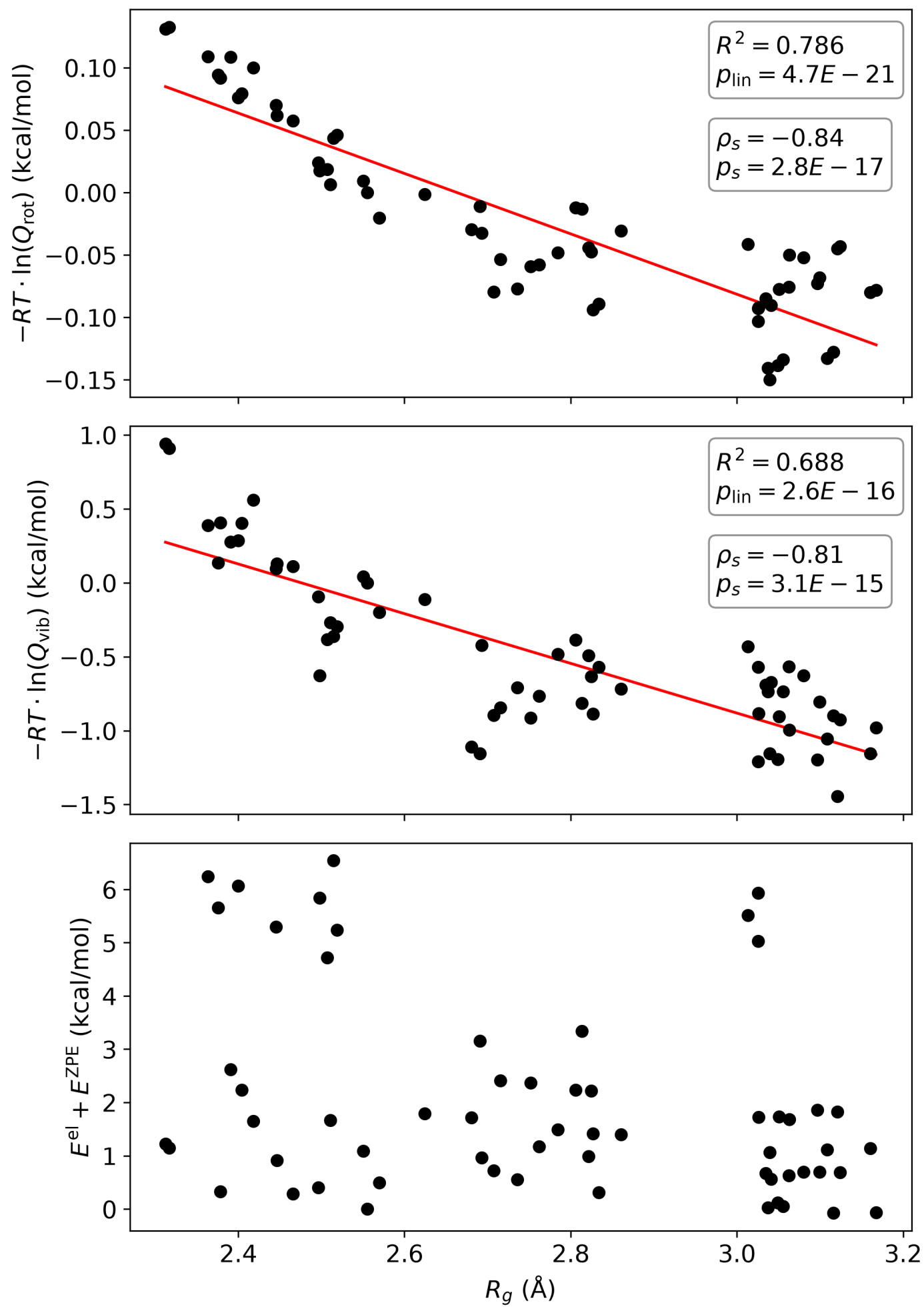

Figure S14: Contributions to the relative Gibbs free energy at 310 K as a function of R_g for geraniol: rotational term (top), vibrational term (middle), and zero-point-inclusive electronic energy (bottom). All quantities are referred to the conformer of lowest electronic energy, so that the three contributions yield $G_{\rm rel}(310)$.

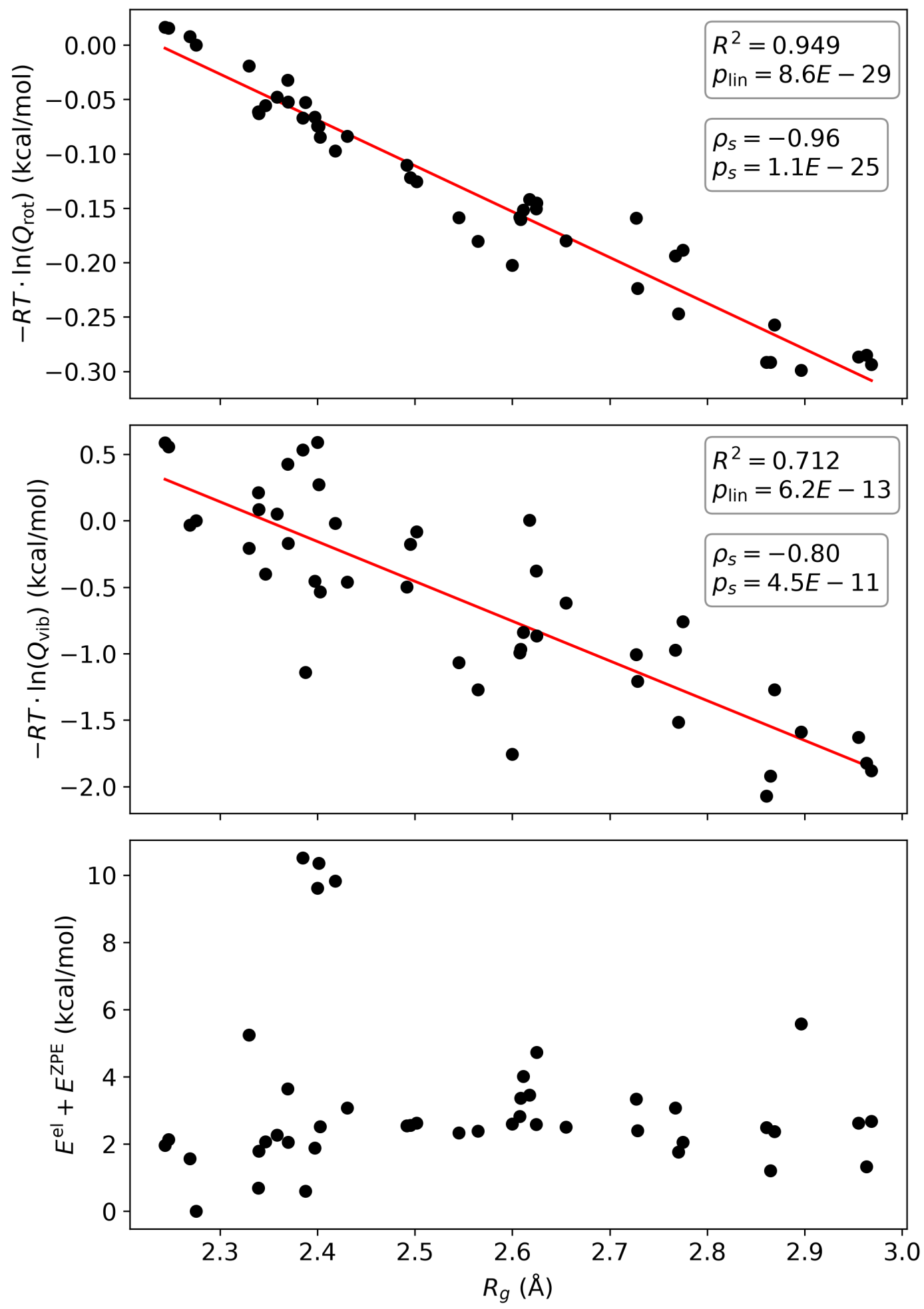

Figure S15: As in Fig. S14, for nerol.

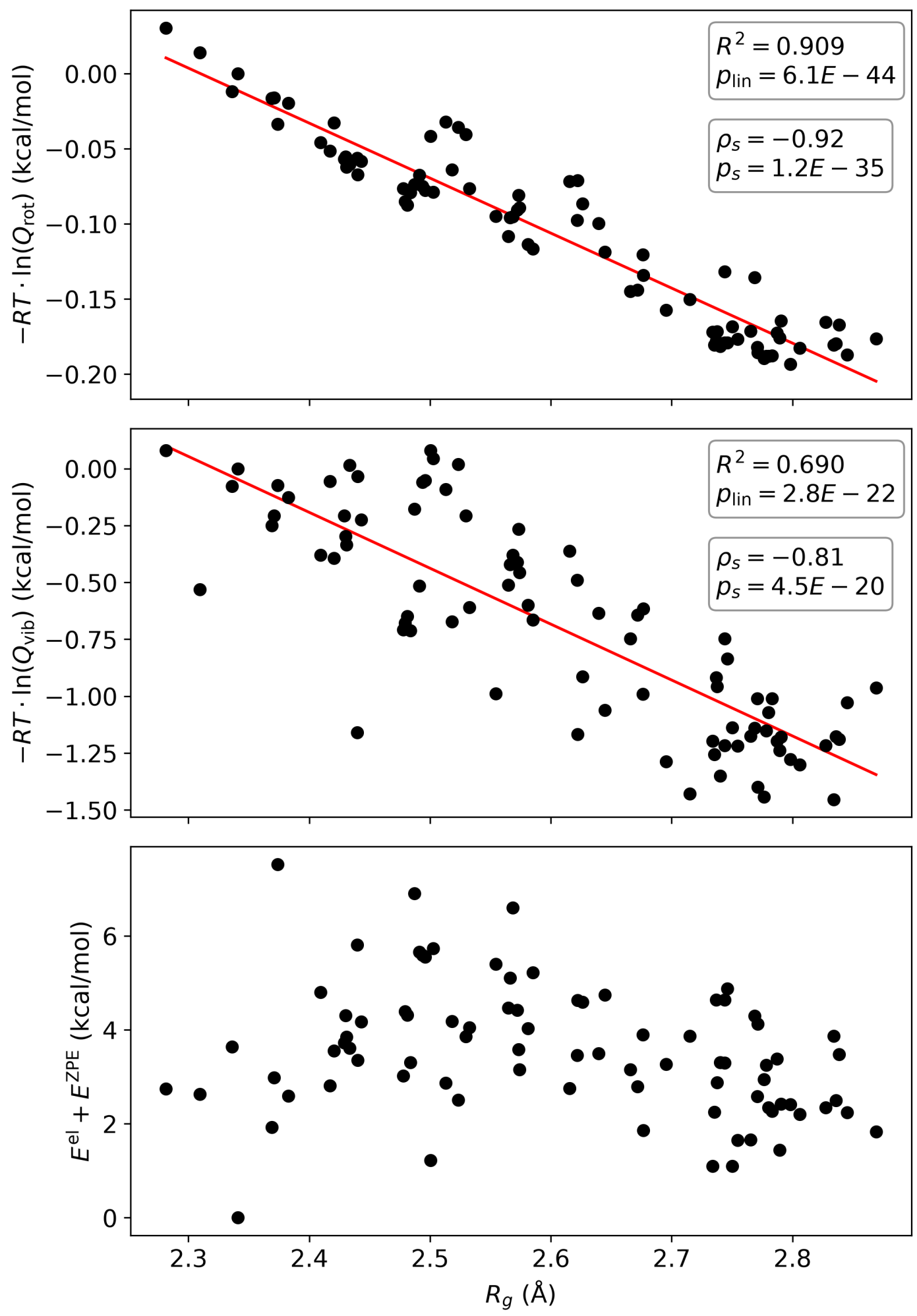

Figure S16: As in Fig. S14, for linalool.

S7. Determination of the number of conformational families

The clustering analyses were performed in the reduced PCA space. In the three systems the first three principal components account for more than 80% of the descriptor variance (84%, 86% and 82%, respectively) and define the space in which all partitions were obtained.

Partitions with $k = 2$ to 20 groups were examined. Every model was fitted from 50 random starting points, and the whole procedure was repeated with ten independent random seeds. For each k the solution optimizing the objective function of the corresponding method (inertia for K-means, BIC for the GMMs) was retained, and the reproducibility of the resulting partition was quantified through the adjusted Rand index (ARI) computed against the remaining nine seeds. All calculations were carried out with `scikit-learn` (version 1.9.0).

S7.1. Assessment of K-means partitions

Fig. S17 shows the silhouette coefficient and the Davies–Bouldin index as a function of the number of clusters (k) for the K-means partitions. Higher silhouette and lower Davies–Bouldin values indicate more compact and better separated clusters.

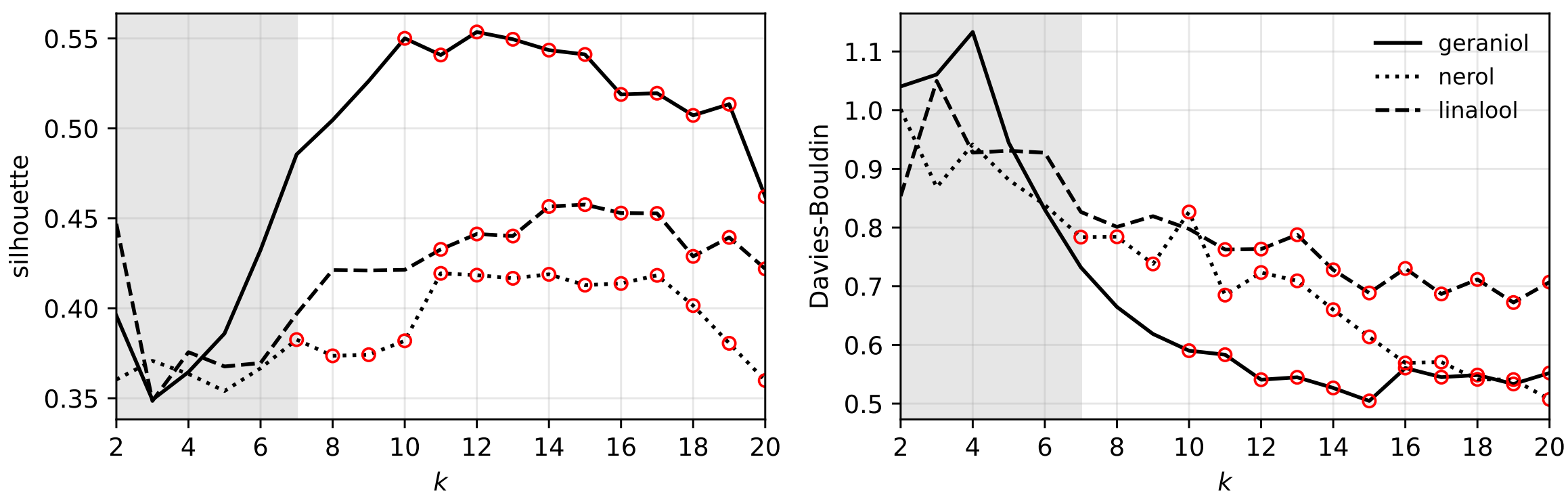

Figure S17: Silhouette (left) and Davies–Bouldin (right) scores as a function of the number of clusters for the K-means partitions. Open red circles mark values of k for which the smallest cluster contains three structures or fewer. The shaded region marks the contiguous range from $k = 2$ over which the partitions remain highly reproducible across initializations ($\mathrm{ARI} \geq 0.95$).

Neither criterion identifies a well-defined optimum number of clusters. The Davies–Bouldin index decreases almost monotonically, whereas the silhouette coefficient shows only shallow maxima at large k, where the smallest clusters contain one to three structures. Reproducibility is high ($\mathrm{ARI} \geq 0.95$) only up to $k \simeq 7$ when the three systems are considered together, nerol being the limiting case; at larger k it becomes irregular, and apparently stable solutions may simply reflect partitions containing single-structure clusters.

Overall, the K-means analysis does not provide evidence for a well-defined partition of the conformational space into discrete clusters. Since K-means assigns each structure to a single cluster on the basis of Euclidean distance and is best suited to relatively compact and well-separated groups, we subsequently examined Gaussian mixture models (GMMs). The probabilistic GMM description allows partially overlapping regions and, through the covariance matrix, a more flexible representation of their geometry in PCA space.

S7.2. Gaussian mixture models and BIC profiles

GMMs were evaluated to account for possible anisotropic or partially overlapping conformational distributions. The representation used in the main text employed a common general covariance matrix for all components (`covariance_type="tied"`), so that the components have the same shape, size, and orientation in the reduced PCA space and differ only in their position. This regularization is consistent with the use of the GMM as a coarse-grained partition of a continuous descriptor distribution rather than as a set of intrinsically distinct structural families. For comparison, the alternative `full`, `diag`, and `spherical` covariance parametrizations were also tested. The corresponding BIC profiles are compared in Fig. S18.

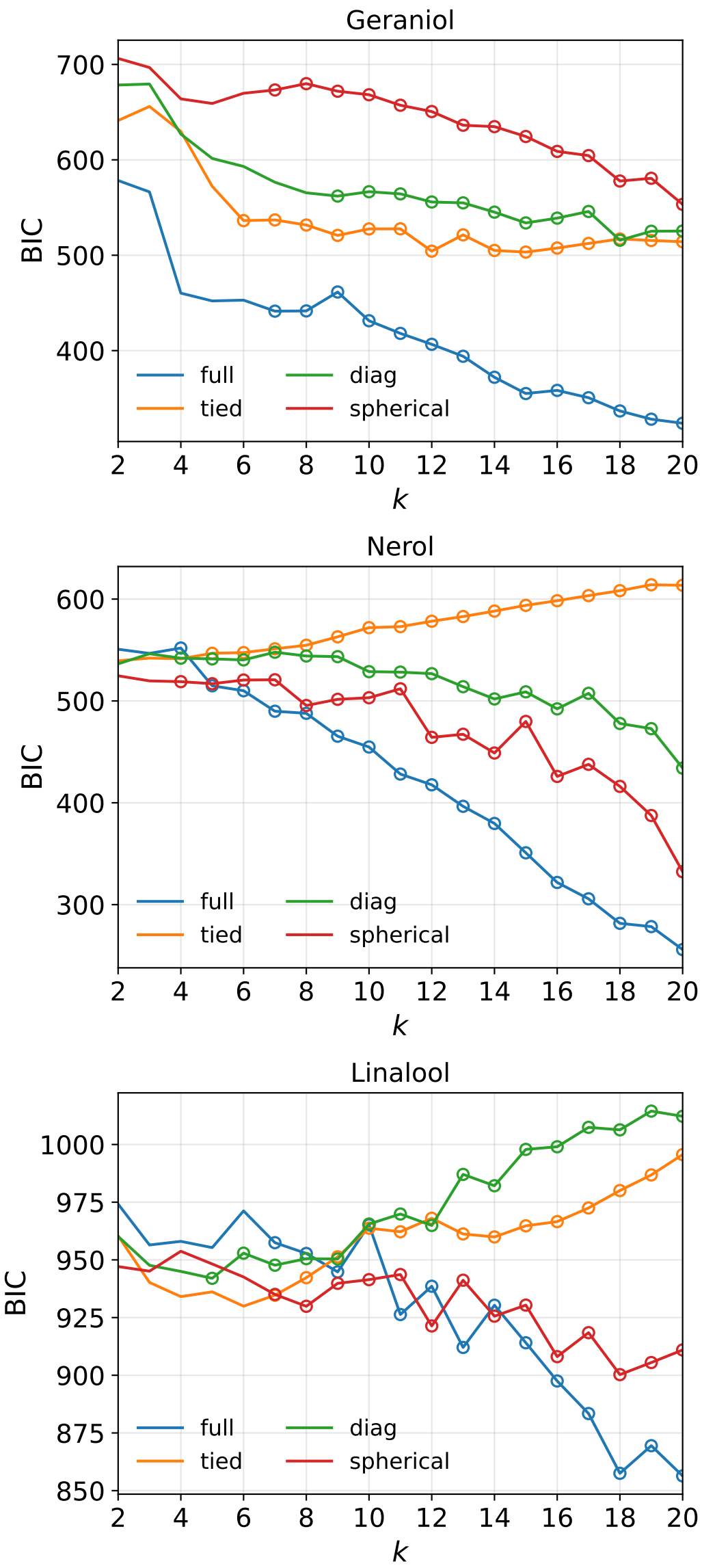

Figure S18: BIC as a function of the number of GMM components for the four covariance parametrizations. Open circles mark k values with components of three structures or fewer.

Because the BIC balances goodness of fit against model complexity, lower values indicate a preferred model, and a well-defined minimum would provide a statistical criterion for selecting the number of GMM components.

When each component is assigned its own covariance parameters (`spherical`, `diag`, and `full`), the BIC generally decreases as k increases, with the lowest values occurring at relatively large numbers of components. In this region, single-structure components already appear, indicating that the improved fit is associated with an increasingly fragmented partition. In nerol, the most extreme case, the BIC of the `full` model falls from 550.8 at $k = 2$ to 255.9 at $k = 20$.

This behavior reflects the increasing flexibility of these models relative to the limited sample size. For example, in the three-dimensional PCA space used here, the covariance matrix of each `full` component is

$$\mathbf{\Sigma} = \begin{pmatrix} \sigma_{11} & \sigma_{12} & \sigma_{13} \\ \sigma_{12} & \sigma_{22} & \sigma_{23} \\ \sigma_{13} & \sigma_{23} & \sigma_{33} \end{pmatrix},$$

and therefore contains six independent covariance parameters, since the covariance matrix is symmetric ($\sigma_{ij} = \sigma_{ji}$), in addition to three parameters defining the component mean. The mixture model also includes $k - 1$ independent component weights. Thus, for $k = 4$, a `full` GMM already contains $4 \times 6 = 24$ covariance parameters and $24 + 4 \times 3 + (4 - 1) = 39$ independent parameters in total, for datasets comprising only 44–83 structures. As k increases, the model becomes increasingly prone to overfitting, with improvements in likelihood arising from fitting progressively smaller groups of structures rather than from identifying well-populated conformational families.

Accordingly, to reduce the tendency toward overfitting, we focus on the `tied` parametrization, in which all components share a common covariance matrix. This constraint substantially reduces model flexibility, although it still does not reveal a consistent optimum. The BIC increases overall with k in nerol, reaches a shallow minimum at $k = 6$ in linalool, and decreases slowly in geraniol, where its lowest value occurs at $k = 15$ and the smallest component contains only two structures. Thus, the preferred number of components is strongly system-dependent and no common optimum is identified across the three terpenoids.

Overall, the BIC analysis does not identify a consistent and well-supported number of conformational families across the three systems.

S7.3. Choice of the number of components

Since no statistical criterion identifies a unique number of conformational families, the GMM partition used in the main text was selected as an operational coarse-grained representation rather than as an optimal model. The choice of $k = 4$ represents a compromise between reproducibility, population of the resulting regions, and preservation of thermodynamically distinct conformational behavior. Partitions with fewer components ($k = 2$–3) merge regions with distinct thermodynamic characteristics, whereas increasing k beyond four introduces very small components and increasingly irregular reproducibility.

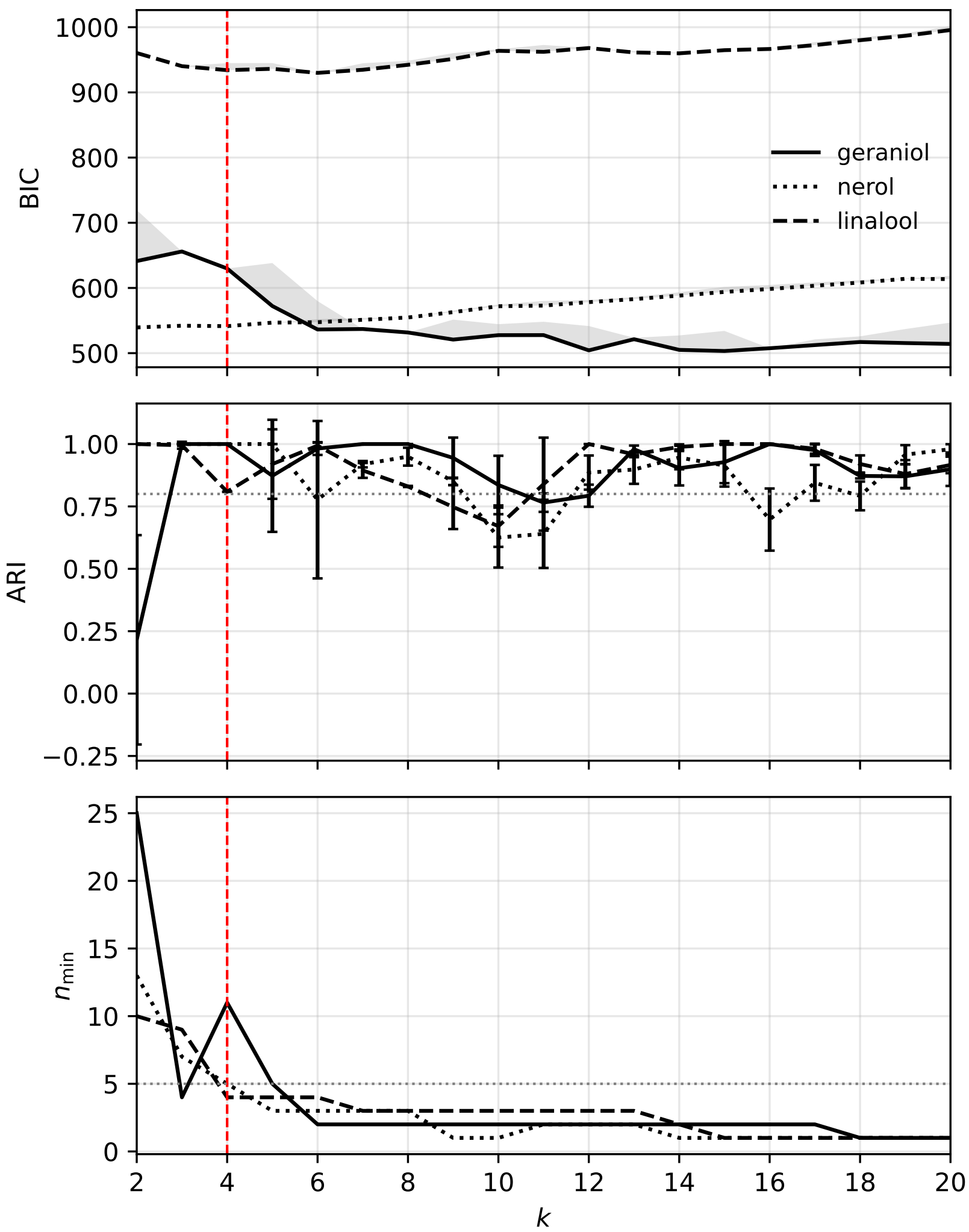

Figure S19: BIC (upper panel), adjusted Rand index against independent initializations (middle panel) and size of the smallest component (lower panel) as a function of the number of components, for GMMs with a common covariance matrix. The shaded band in the upper panel indicates the spread of the BIC among seeds. The vertical dashed line marks the partition adopted in the main text.

At $k = 4$, the smallest component contains 11, 5, and 4 structures for geraniol, nerol, and linalool, respectively. The partitions are exactly reproducible in geraniol and nerol (ARI $= 1.00$), whereas linalool shows lower stability (ARI $= 0.81$). Importantly, $k = 4$ is the largest common partition for which all three systems retain more than three structures in every component (see also open circle marks in Fig. S18). At $k = 5$, nerol already contains a three-structure component and the geraniol partition becomes less stable (ARI $= 0.87$); further increases in k lead to increasingly fragmented partitions and irregular reproducibility.

A three-component representation provides a coarser description (Table S5). In linalool, two regions that at $k = 4$ have median relative Gibbs free energies of 1.55 and 3.72 kcal mol^{-1} are merged into a single region with a median of 2.72 kcal mol^{-1}, thereby losing a thermodynamic distinction between conformational regions of comparable global extension. In geraniol, the two extended regions obtained at $k = 3$ are nearly indistinguishable in global shape ($\langle R_g \rangle = 2.79$ and 2.80 Å; $\langle d_{\mathrm{O-C_{far}}} \rangle = 7.50$ and 7.68 Å), while the compact region contains only four structures. In nerol, the compact region with the lowest mean electronic energy identified at $k = 4$ is no longer resolved. Thus, $k = 3$ provides a coarser representation that removes chemically relevant distinctions.

Table S5: Summary of the representative conformational regions obtained from the three-component GMM representation. N is the number of conformers assigned to each region. Median relative Gibbs free energies at 310 K and interquartile ranges are reported, with the IQR defined as the range between the first and third quartiles. Energies are given in kcal mol^{-1} and distances in Å.

System	Region	N	$\langle R_g \rangle$	$\langle d_{\mathrm{O-C_{far}}} \rangle$	$\langle E_{\mathrm{rel}} \rangle$	Median $G_{\mathrm{rel}}(310)$	IQR($G_{\mathrm{rel}}(310)$)
Geraniol	Compact	4	2.36	4.81	2.64	3.72	3.48–4.86
	Extended I	25	2.79	7.50	1.93	1.49	0.96–2.32
	Extended II	31	2.80	7.68	2.58	1.97	1.62–3.36
Nerol	Compact	24	2.39	5.39	3.72	3.09	2.49–4.03
	Intermediate	7	2.61	6.30	3.64	3.23	2.86–4.16
	Extended	13	2.83	7.75	3.07	1.84	1.14–2.69
Linalool	Folded	9	2.42	4.44	2.72	3.00	2.37–3.39
	Intermediate	31	2.61	6.33	3.24	2.72	1.29–3.57
	Extended	43	2.64	6.42	4.29	3.54	2.22–4.40

The four-component representation was therefore retained as an operational coarse-grained description: it provides greater structural and thermodynamic resolution than $k = 3$, while avoiding the small components and increasing instability that begin to appear at larger k. This choice does not imply the existence of four discrete conformational states.
